# Enhancement of hemostatic properties of *Cyclotella cryptica* frustule through genetic manipulation

**DOI:** 10.1186/s13068-023-02389-x

**Published:** 2023-09-14

**Authors:** Lulu Wang, Yan Sun, Ruihao Zhang, Kehou Pan, Yuhang Li, Ruibing Wang, Lin Zhang, Chengxu Zhou, Jian Li, Yun Li, Baohua Zhu, Jichang Han

**Affiliations:** 1 Key Laboratory of Mariculture, Ministry of Education, Ocean University of China, Qingdao, 266003 China; 2 College of Food and Pharmaceutical Sciences, Ningbo University, Ningbo, 315200 China; 3 Laoshan Laboratory, Qingdao, 266237 China; 4 Department of Marine Organism Taxonomy and Phylogeny, Institute of Oceanology, Chinese Academy of Sciences, Qingdao, 266071 China; 5 State Key Laboratory of Quality Research in Chinese Medicine, Institute of Chinese Medical Sciences, University of Macau, Macau, 999078 China; 6 Key Laboratory of Applied Marine Biotechnology, School of Marine Sciences, Ningbo University, Ningbo, 315200 China; 7 School of Biological and Chemical Engineering, Panzhihua University, Panzhihua, 617000 China

**Keywords:** Diatom frustule, Silica, Morphology remolding, Genetic modification, Hemostatic material

## Abstract

**Background:**

The silicified cell wall of diatoms, also known as frustule, shows huge potential as an outstanding bio-nanomaterial for hemostatic applications due to its high hemostatic efficiency, good biocompatibility, and ready availability. As the architectural features of the frustule determine its hemostatic performance, it is of great interest to develop an effective method to modify the frustule morphology into desired patterns to further improve hemostatic efficiency.

**Results:**

In this study, the gene encoding Silicalemma Associated Protein 2 (a silicalemma-spanning protein) of *Cyclotella cryptica* (*CcSAP2*) was identified as a key gene in frustule morphogenesis. Thus, it was overexpressed and knocked down, respectively. The frustule of the overexpress lines showed no obvious alteration in morphology compared to the wild type (WT), while the size, specific surface area (BET), pore volume, and pore diameter of the knockdown strains changed greatly. Particularly, the knockdown frustules achieved a more pronounced coagulation effect and in vivo hemostatic performance than the WT strains. Such observations suggested that silicalemma proteins are ideal genetic encoding targets for manipulating frustule morphology associated hemostatic properties. Furthermore, the Mantel test was adopted to identify the key morphologies associated with *C. cryptica* bleeding control. Finally, based on our results and recent advances, the mechanism of frustule morphogenesis was discussed.

**Conclusion:**

This study explores a new strategy for enhancing the hemostatic efficiency of the frustule based on genetic morphology modification and may provide insights into a better understanding of the frustule morphogenesis mechanism.

**Supplementary Information:**

The online version contains supplementary material available at 10.1186/s13068-023-02389-x.

## Background

Uncontrollable hemorrhage accounts for approximately 50% of deaths in military and 15–25% of deaths in civilian trauma [1, 2]. Rapid control of bleeding is imperative to improve the survival rate. However, traditional hemostatic agents, including both polymer and inorganic materials, have presented various disadvantages (*e.g*., the exothermic reaction of QuikClot^®^ and the allergic reaction caused by HemCon^®^) and limitations over the past decades [3–5]. Consequently, it is imperative to develop novel hemostatic agents with high efficiency and minimal side effects [6].

Due to its high liquid absorbability (conferred by its large specific area, porous surface, and internal hollow microstructure) and negatively charged surface (mainly contributed by its rich silanol groups) that can initiate the intrinsic coagulation pathway and activate the coagulation cascade by interacting with positively charged clotting proteins (*e.g.*, coagulation factor XII), mesoporous silica has been considered as one of the most promising novel inorganic materials suitable for hemostasis applications [5, 7]. However, the fabrication procedure of mesoporous silica is costly, time-consuming, and requires toxic substances, which limits its widespread utilization [8]. Diatoms, one of the most abundant groups of eukaryotic microalgae, contribute to nearly half of the oceanic primary production and are famous for their high biomass and highly ornamented, silicified and porous cell wall (termed as frustule). Indeed, frustules, mainly made up of amorphous hydrated silica, are outstanding mesoporous biosilica materials. In addition to the characters stated above (*i.e*., high absorbability and negatively charged surface), frustules also exhibit additional advantages, such as favorable biocompatibility, good biodegradability, and easy availability [9].

In recent years, some reports have illustrated the potential of various frustules extracted from different diatom species as outstanding hemostasis agents [10, 11]. Moreover, several efforts have been made to enhance the hemostatic performance and/or biocompatibility further. For example, the addition of calcium chloride into the culture medium successfully integrated calcium (coagulation factor IV) into the biosilica, generating Ca-biosilica. Both the clotting time and blood loss of Ca-biosilica were significantly shorter and lower than those of Ca-free biosilica [12]. In vitro immobilization of chitosan and dopamine has also been successfully adopted to enhance the biocompatibility of the frustule [13–15]. Besides surface functionalization and particle immobilization, morphology modification is an alternative strategy to enhance the hemostatic efficiency of frustule because morphological features such as specific surface area (BET), cell size, and pore diameter are closely associated with hemostatic performance [10, 11]. Several methods are currently available to alter frustule morphology. For example, hydrofluoric acid (HF) etching can enlarge the pore diameter and reduce the thickness of frustule. However, this method can lead to a decline in BET and adsorption capacity, negatively affecting hemostasis performance [16]. Modulating cultivation conditions including salinity [17, 18], light wavelength [19, 20] and temperature [21], can also result in the alteration of morphological features of frustule. However, the relationship between abiotic factors and induced morphological alterations is still ambiguous [22], and related research cannot currently provide accurate guidance for desirable frustule production.

Genetic manipulation is a promising approach for remodeling frustule microstructure, but the understanding of frustule morphogenesis is still in its early stages. Only a limited number of protein families, such as silaffins [23–25], silacidins [26, 27], silicanins (SiMats) [28], SAPs [29], cingulins [30], and pleuralins [31], have been identified as being associated with silica polymerization. Recently, De Haan et al. used live-cell confocal microscopy and advanced electron microscopy to observe the frustule assembly process for the first time and confirmed that the frustule forms inside a membrane (called the silicalemma)-bound compartment (Silica Deposition Vesicle, SDV). The authors also found that the silicalemma tightly surrounds the forming frustule during the whole silicification process, suggesting an essential role of the silicalemma in frustule morphology [32]. Based on previous observations that the cytoskeleton plays an important role in shaping frustule, it can be speculated that silicalemma-spanning proteins mediate the interaction between the SDV and cytoskeleton [28, 29, 33]. Thus, genetic manipulation of the genes encoding silicalemma-spanning proteins is likely to alter the frustule morphology. Among all known silica-associated proteins, SAPs and Silicanin-1 (Sin1) have been confirmed as silicalemma-spanning proteins (additional seven silicalemma proteins have been proposed by Kröger team recently, but further identification is still needed) [28, 29, 34]. Two teams, Hildebrand and Kröger, have conducted pioneering attempts in *Thalassiosira pseudonana* recently and found that knockdown of *TpSAP1* and *TpSAP3* as well as knockout of *TpSin1*, resulted in changes in surface microstructures and biosilica content of frustules, confirming the important role of silicalemma-spanning proteins in frustule morphology. Although the characters essential for hemostasis performance (such as frustule size, pore diameter, and pore density) showed no visible differences between the wild type (WT) and mutants, these studies provided a solid foundation for further exploration [29, 35].

**Scheme 1.**
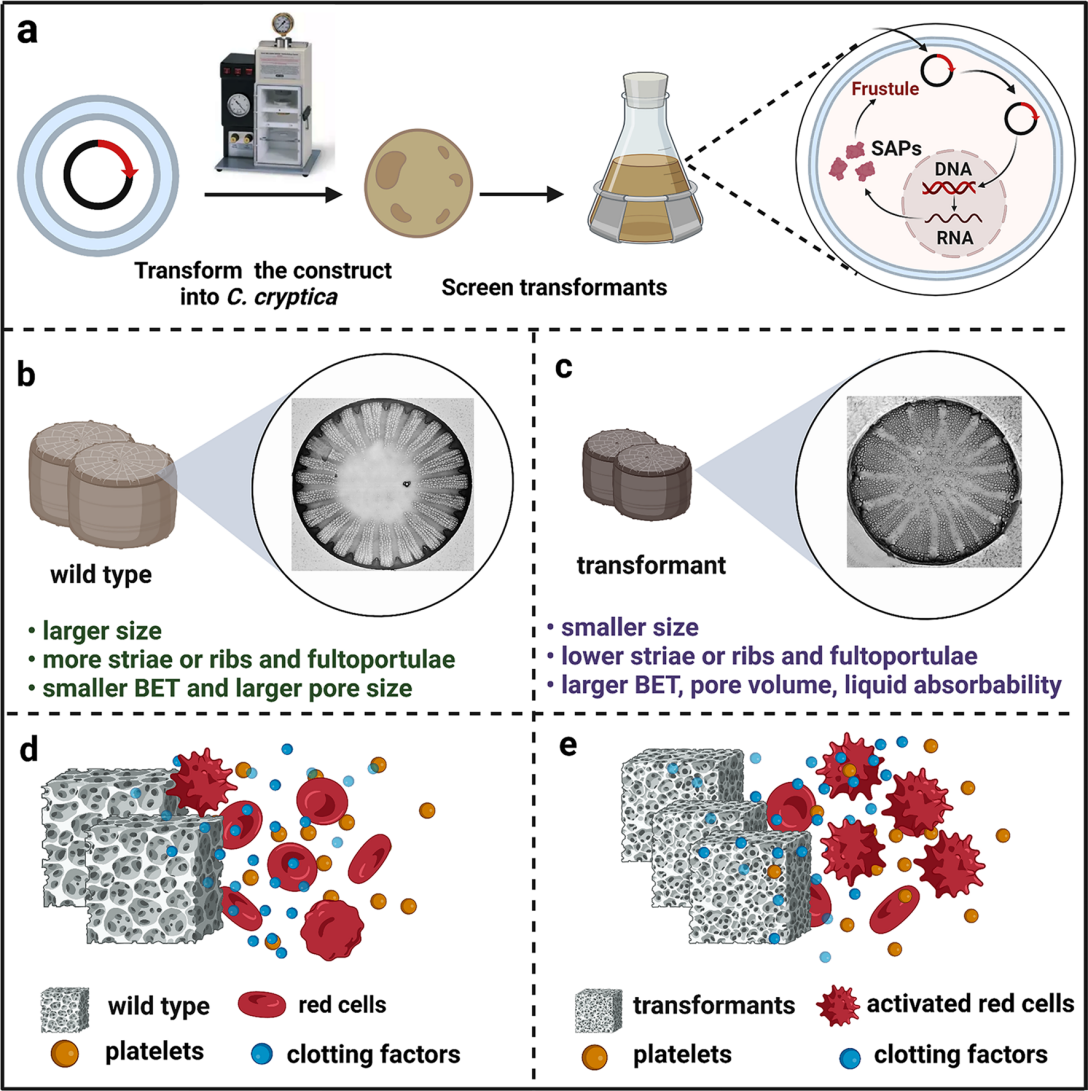
Preparation of genetically manipulated *C. cryptica*. **a** Acquisition of transformants; **b** and **c** represent the surface pattern and physical features of WT and *CcSAP2* knockdown strains, respectively; **d** and **e** show the hemostatic performance of the frustule is enhanced by genetic manipulation based on *CcSAP2*

*Cyclotella cryptica* is an emerging model diatom species that has attracted abundant attention for commercial applications due to its high growth rate and rich fucoxanthin content. Recent studies have also demonstrated that the frustule of *C. cryptica* possesses outstanding hemostatic performance and favorable biocompatibility. Therefore, it is meaningful to verify whether genetic manipulation strategies are feasible to enhance the hemostasis performance of *C. cryptica* frustule. In this study, we first investigated the transcript patterns of three *CcSAPs* in response to silicate starvation and replenishment conditions, and the results suggested that *CcSAP2* may function in the early stage of frustule formation. Subsequently, we used overexpression and knockdown to further identify the impact of *CcSAP2* on biosilica morphogenesis and to investigate the feasibility of modifying frustule morphologies by genetic engineering methods. Interestingly, the overexpression lines exhibited a similar morphology phenotype to the WT, while the antisense clones showed quite different frustule characters. We then systematically tested the physicochemical properties, hemostatic performance (both in vitro and in vivo), and the biocompatibility of *CcSAP2* knockdown frustules (Scheme 1). To identify the key physicochemical properties underlying hemolytic activity, we further adopted the Mantel test to determine the correlation between physiochemical characters and hemostasis performance. Additionally, we discussed the functional mechanism of SDV transmembrane proteins on frustule morphogenesis.

## Method and materials

### Diatom species, culture conditions, and growth performance

*Cyclotella cryptica* was obtained from the Lab of Applied Microalgae Biology at the Ocean University of China and was grown in f/2 medium at 21°C [36]. The culture was maintained under a light intensity of 40 µmol photons m^2^ s^–1^ and a light/dark rhythm of 12/12 h. During the batch cultivation, the cell density (optical density at 750 nm, OD_750_) was measured daily using a U-3310 spectrophotometer (Hitachi, Tokyo, Japan). The specific growth rate was calculated using Eq. (1):

1 $$\mu \, = \,({\text{LnN}}_{{\text{t}}} {-}{\text{LnN}}_{0} )/\Delta {\text{t,}}$$

where N_t_, N_0_, and Δt represented the maximum density, initial density, and the entire cultivation period (in days). On the final day of cultivation, the cell size was determined by surveying 100 cells of each strain using a microscope with 400 times magnification.

### Sequence analysis and key CcSAP determination

The Hildebrand group’s research [29] was used as a basis for conducting BLASTp searches of *CcSAP1*–*3* (JGI protein ID: 16341, 11599, 12802) against the genomes *of Fragilariopsis cylindrus*, *Fistulifera solaris*, *Minidiscus variabilis*, *Nitschia inconspicua*, *Phaeodactylum tricornutum* (V2.0), *Pseudonitzschia multiseries*, *Seminavis robusta*, *Thalassiosira oceanica*, and *T. pseudonana* (v3.0) in the JGI database to identify homologous SAP sequences. SignalP-5.0 [37], DeepTMHMM 1.0.18 [38], InterPro [39], and NetPhos-3.1 [40] bioinformatics tools were then used to identify signal peptides, transmembrane domains, cytoplasmic domains, and post-translational modification sites.

The transcriptional patterns of *CcSAP1*–*3* were examined using qRT-PCR. Prior to RNA extraction, *C. cryptica* cells were synchronized using silicon starvation for 48 h. Cells were collected by centrifugation (3000*g* for 5 min) at seven different timepoints, with four during silicon starvation and three after silicon replenishment. RNA was treated with DNase and purified using the Plant RNA Kit (OMEGA, USA), and equal amounts of RNA were used to synthesize cDNA using SuperScript III reverse transcriptase (Vazyme, China). qRT-PCR was performed using a QuantStudio 5 (ABI, USA) and a PowerUp SYBR Green Master Mix (ThermoFisher Scientific, USA), with *histone H4* serving as the internal reference gene. Primers were designed using Primer Premier 5.0 and are listed in Additional file 1: Table S1.

### Vector construction and genetic manipulation

To enable genetic transformation of *C. cryptica*, we constructed the plasmid CcpPha-T1 by modifying pPha-T1. To ensure that expression was controlled by the native promoter/terminator cassette, we amplified the promoter (587 bp, – 1 to – 587 bp upstream of ATG) and terminator (999 bp, 1 to 999 bp downstream of TAG) of *CcSIT1* (JGI protein ID: 13652) from *C. cryptica* genomic DNA. We then inserted these fragments between the *Nde* I-*EcoR* I and *Sph* I-*Hind* III sites of pPha-T1, replacing the fcpA promoter and terminator (see Additional file 1: Fig. S1).

For the overexpression vector, the coding region of *CcSAP2* (642 bp) was amplified from complementary DNA using primers SAP2-s-fw (containing a *Sac* I site) and SAP2-s-rv (containing a *BamH* I site). After digestion with *Sac* I and *BamH* I, the amplicon was cloned into CcpPha-T1 (between *Sac* I-*BamH* I sites) to generate the final overexpression vector (CcpPha-SAP-OE). To perform RNA interference, the antisense fragment of *CcSAP2* (639 bp) was amplified using SAP2-as-fw (containing a *BamH* I site) and SAP2-as-rv (containing a *Sac* I site). After digestion with *Sac* I and *BamH* I, the amplicons were cloned into *Sac* I-*BamH* I sites of CcpPha-T1, generating the final knockdown vector named CcpPha-SAP-AS. The map of CcpPha-T1 is shown in Additional file 1: Fig. S1, and the primers used for vector construction are listed in Additional file 1: Table S1.

The tungsten particles were prepared following the instructions of the Bio-Rad Biolistic PDS-1000/He manual (BIORAD, Hercules, CA, USA). Cells in the exponential growth phase (OD_750_ of 0.15) were collected by centrifugation (3000*g*, 5 min) and concentrated to 5 × 10^8^ cells mL^–1^ using liquid f/2 medium. Approximately 2.5 × 10^8^ cells were plated onto the center of solid f/2 medium (1% agar, without antibiotics) for subsequent bombardment, as previously described [41, 42]. After bombardment, the plates were incubated for 48 h under continuous white fluorescent light (30 μmol photons m^–2^ s^–1^). The cells were then washed off and transferred to solid f/2 medium (1% agar) containing 0.1 μg mL^–1^ Zeocin^®^ for cultivation under constant light (40 μmol photons m^–2^ s^–1^) [43]. Visible algal colonies were picked after approximately four weeks and transferred to liquid f/2 medium for further PCR screening.

Five hundred microliters of cells (OD_750_ of 0.15), collected by centrifugation (3000*g*, 5 min), were resuspended in 100 µL of lysis buffer (Plant Direct PCR Kit, Vazyme, China) and incubated at 95°C for 15 min to extract genomic DNA. After centrifugation, specific primers Ble-fw and Ble-rv (listed in Additional file 1: Table S1) were used to amplify the bleo gene from one microliter of supernatant, and the amplicons were further sequenced.

### RNAi efficiency analysis and silicon incorporation assay

The RNA extraction and qRT-PCR procedures were the same as described in the section of “Sequence analysis and key CcSAP determination” section. The primers used are listed in Additional file 1: Table S1. The WT strain and the empty CcpPha-T1 transformed strain (designated as NEP) were used as negative controls.

Cells in logarithmic growth phase, grown in f/2 medium, were harvested under sterile conditions by centrifugation at 3000*g* for 5 min. After being washed twice with silicate-free artificial sea water (ASW), collected cells were inoculated in silicate-free ASW medium and concentrated to a density of 2.5 × 10^5^ cells mL^–1^. After 48 h of silicate starvation, PDMPO ([2-(4-pyridyl)-5-((4-(2-dimethylaminoethylamino) methoxy) phenyl) oxazole]) was added to the medium at a final concentration of 100 ng mL^–1^. After 5 min, sodium silicate was added to a final concentration of 25 μM to initiate the cell cycle and cell wall formation procedure [29, 44, 45]. At four time points (30, 60, 90, and 120 min), cells were collected, washed twice with silicon-free ASW, and their fluorescence intensities (concentrated to the same density in advance) were investigated via an flow cytometry (Beckman Coulter, USA) using excitation at 488 nm and emission at 530 nm.

The biosilica content of the diatom cell wall was quantified using the silicomolybdate assay according to previous studies [27, 35, 46]. In brief, 10^6^ cells were collected by centrifugation (3000*g*, 5 min), washed twice with MilliQ water, and then resuspended in 100% methanol. The cells were vortexed until they became colorless and were then collected, washed, and transferred into 1 mL MilliQ water. Next, 500 µL of NaOH solution (6 M) were added to dissolve the silica, and the mixture was incubated at 95°C for 1 h. After centrifugation, the supernatant was collected to determine the silicic acid content. Briefly, 25 µL of the above supernatant was mixed with 100 µL MilliQ water and 50 µL molybdate reagent. The mixture was incubated at 25°C for 10 min, and then 75 µL of reducing agent were added and incubated at 25°C for 3 h. For each sample, the absorbance at 810 nm was measured using a microplate reader (Synergy HT, BioTek, USA), and this measurement was used for further quantification of the biosilica content based on a standard curve prepared using sodium hexafluorosilicate.

### Preparation of diatom frustules

All strains of *C. cryptica* for frustule preparation were cultivated under the same conditions. Briefly, 500 mL of *C. cryptica* cells were collected by centrifugation (3000*g*, 5 min), and then the pellets were rinsed using MilliQ water to remove the salt and other unnecessary components. After another centrifugation (5000*g*, 5 min), 20 mL of sulfuric acid were mixed with the pellets, and then the mixture was heated at 60°C for 1 h. After being left at room temperature for 24 h, 20 mL of nitric acid were added to the mixture and heated at 60°C until the mixture became colorless. The cleaned frustules were finally collected using sieves, washed with ethanol, and then dried for later use.

### Determination of diatom frustules characteristics

For the preliminary determination of cell size, 100 cells of each strain were randomly measured using light microscope (Nikon, Japan) under 400-fold magnification. The detailed morphology characteristics were further determined using a scanning electron microscope (SEM, JSM-6010LA, JEOL, Japan), and the frustules were purified using the method same with our previous report [10]. The surface elements and functional groups were determined using energy dispersive X-ray spectroscopy (EDXS, SEM, JSM-6010LA, JEOL, Japan) and Fourier transform infrared spectroscopy (FTIR, 5700, Nicolet, USA). Zeta potential was measured by A Malvern Zetasizer (Zetasizer Nano, Malvern, England) in phosphate-buffered solution (PBS) at pH 7.4. The liquid absorption capability was tested using simulated body fluid (SBF) with the methods same with previous studies [13], and the absorption ratio (AR) was calculated using Eq. (2):

2 $${\text{AR}}\% \, = \,({\text{W}}_{{{\text{wet}}}} {-}{\text{W}}_{{{\text{dry}}}} ) \, /{\text{ W}}_{{{\text{dry}}}} \, \times \,{1}00,$$

which W_wet_ and W_dry_ represent the weights of the wet and dry frustules. N_2_ adsorption desorption isotherm was carried out using automatic gas adsorption analyzer (ASAP 2460, Micromeritics, USA) to calculate the BET, pore diameter, and pore volume of frustules.

### In vitro blood clotting assessment hemostatic mechanism

In total, four types of *C. cryptica* frustules (three antisense clones and one WT strain) were used to conduct whole blood clotting tests based on New Zealand white rabbit blood (3.8% sodium citrate:blood = 1:9). The clotting time was recorded until the blood stopped flowing completely. After coagulation, blood clots were rinsed with phosphate buffer solution (pH 7.4) three times, and then immobilized with 2.5% glutaraldehyde at 4°C for 2 h. After being dehydrated using gradient alcohol and dried using critical point drier, the blood cells were further observed using SEM. The plasma coagulation properties of four types of frustules were investigated using a semiautomatic coagulation analyzer (TS6000, MD PACIFIC, China). The prothrombin time (PT) and activated partial thromboplastin time (aPTT) were determined using corresponding assay kit (MD PACIFIC, China) [10, 15]. The whole blood coagulation dynamics were determined based on a Thromboelastography (TEG) analyzer (TEG^®^5000, Haemonetics Corporation, USA), and four parameters including reaction time (R, min), angle α (α, degree), clot formation time (K, min), and maximum amplitude (MA, mm) were chosen for the evaluation of coagulation behavior. In all of these tests, pure re-calcified blood was used as the blank control, and commercial QuikClot^®^ was used as the positive control.

### In vivo hemostatic assessment

Both rat tail amputation and rat femoral artery models were used to evaluate the in vivo hemostatic performance. For the former model, the tails of anesthetized Sprague–Dawley (SD) rats (weight of 220 ± 20 g) were cut to 50% of their length using surgical scissors. After being exposed to air for 15 s to ensure normal blood loss, the injured tails were placed in 100 mg of frustule or QuikClot^®^. The clotting time and blood loss were recorded during the hemostatic process [11]. For the latter model, when the rat femoral artery in the right hind limb was cut with a scalpel, 50 mg of frustule or QuikClot^®^ was immediately poured onto the injury. The clotting time and blood loss were recorded when the wound stopped bleeding thoroughly [47, 48]. In both models, gauze and QuikClot^®^ were used as negative and positive control groups, respectively.

### Biocompatibility evaluation

Biocompatibility was evaluated based on hemolysis rate and cytotoxicity. Different amounts of frustules (0.625, 1.25, 2.5, 5, and 10 mg) were mixed with 1 mL normal saline and maintained at 37°C for 60 min. Then, 20 µL of anticoagulant whole blood (New Zealand white rabbit) were added into the mixture and maintained at 37°C for 60 min. After that, the mixture was centrifuged at 500*g* for 10 min, and the absorbance of the supernatant at 545 nm was determined based on a microplate reader (Synergy HT, BioTek, USA). Distilled water and normal saline without diatom frustules were used as positive and negative control groups. The hemolysis ratio was calculated using Eq. (3):

3 $${\text{Hemolysis ratio }}\left( \% \right)\, = \,({\text{OD}}_{{\text{s}}} {-}{\text{OD}}_{{\text{n}}} ){ /}({\text{OD}}_{{\text{p}}} {-}{\text{OD}}_{{\text{n}}} )\, \times \,{1}00,$$

where D_s_, D_p_, and D_n_ were the absorbance of frustules, distilled water, and saline.

Mouse fibroblast cells (L929) were cultivated in a 96-well plate, each well containing 100 µL of Dulbecco’s modified Eagle’s medium (DMEM). The cell density was concentrated to around 1 × 10^5^ cells per well and incubated for 12 h at 37°C with 5% CO_2_. Subsequently, the initial culture medium of each well was removed and replaced with 100 µL of DMEM containing frustules at various densities (0.625, 1.25, 2.5, 5, and 10 mg mL^–1^). The mixture was then continuously incubated under the same conditions. L929 cells cultivated without frustules were taken as the control. After 24, 48, and 72 h, MTT assays were performed based on an earlier study [49], and the absorbances of the mixture at 490 nm were used to calculate the cell viability using Eq. (4):

4 $${\text{Cell viability }}\left( \% \right)\, = \,{\text{OD}}_{{{\text{test}}}} /{\text{ OD}}_{{{\text{control}}}} \, \times \,{1}00,$$

where OD_test_ and OD_control_ were the optical densities of frustule treatments and the negative control, respectively.

### Correlation analysis between physicochemical properties and hemostasis performance

Aiming to identify the key characteristics determining the hemostasis effect, Mantel test and pairwise Pearson’s correlation analysis were conducted, and the results were visualized using the “LinkET” package of R 4.2.1 software. These analyses were used to investigate the correlation between physicochemical properties (*i.e*., BET, cell size, pore diameter, pore volume, liquid absorbability, zeta potential, and biosilica content) and hemostasis performance (*i.e*., R, K, α, and MA as well as in vivo clotting time and blood loss weight) [47].

### Animal ethics

All animals used in this study were cared for and treated in accordance with the Guidelines for Care and Use of Laboratory Animals of Ocean University of China. All experiments with live subjects were approved by the Instructional Animal Care and Use Committee of the Ocean University of China.

## Results

### Molecular characters of* SAP*s

The lengths of CcSAP1–3 varied within 213–310 amino acids (aa), and the similarities among them were 19.4–22.7%. Though the overall similarity was low, all CcSAPs shared the same sequence features: (1) a signal peptide located at the N-terminal end; (2) a RXL domain (a typical proteolytic cleavage site for biosilica-associated proteins) next to the signal peptide [29]; (3) a long polypeptide region (129–192 aa) contained one or more segments highly rich in serine and glycine (SG-rich domain); (4) a relatively conserved transmembrane domain (the similarities among three *SAP*s were 47.8–65.2%) with lengths varying within 21–23 amino acids; (5) a short C-terminal region (58–68 aa) containing a quite conserved 23 aa polypeptide after the transmembrane domain (Additional file 1: Fig. S2). The N-terminal, accounting for around 2/3 of the total length, was predicted to be inside the SDV, while the C-terminal (approximately 1/4 of the total length) was predicted as cytoplasmic domain (Additional file 1: Fig. S2). Additionally, post-translational prediction tools indicated that CcSAPs (with 32–38 potential phosphorylation sites) were highly phosphorylated (Table 1), which was consistent with other biosilica proteins like silaffins and cingulins, although none of the SAPs showed pentalysine clusters [50].

**Table 1:** Basic characters of different regions of CcSAPs

Name	N-terminal			C-terminal			TM		
	pI	SG (%)	PS	pI	SG (%)	PS	pI	SG (%)	PS
CcSAP1	5.93	38	26	7.74	17.7	5	5.49	13	1
CcSAP2	6.1	34.50	29	9.61	17.2	6	9.7	14.30	3
CcSAP3	4.35	28.10	27	9	13.2	6	5.28	13.6	0

pI, isoelectric point; PS, putative number of phosphorylation sites; TM, transmembrane region; SG indicate the total content of serine and glycine content

Considering that CcSAPs are type 1 transmembrane proteins, we further analyzed their characteristics based on different regions individually (Table 1). In general, the N-terminal sections of all three CcSAPs exhibited much lower pI values, but much higher serine and glycine ratios, as well as more phosphorylation sites than the C-terminal regions. The low pI values of N-terminal regions were consistent with their localization prediction, as the SDV lumen was acidic. As for the high SG ratios and rich phosphorylation sites, they were likely associated with the silica formation activity [28, 29]. Interestingly, the transmembrane regions of the three CcSAPs displayed an unexpected discrepancy in pI values. For *CcSAP2*, its transmembrane region displayed a pI value of 9.7, while those of *CcSAP1* and *CcSAP3* were only 5.49 and 5.28.

The full-length proteins of CcSAP1–3 were used to perform a BLASTp search against the diatom genome databases published in JGI, with an E-value set to 1.0 × 10^−5^. Initially, we assumed that SAPs would be conserved across all diatoms, but we found that only four out of nine diatom species showed ten homologous proteins of CcSAPs (Table 2), namely *F. cylindrus*, *M. variabilis*, *T. oceanica*, and *T. pseudonana*. Since the conservation level varied across different regions, we further conducted BLASTp searches using partial sequences (*i.e.,* N-terminal portion without signal peptide, transmembrane domain, and C-terminal portion) following the same procedure. The results were consistent with those obtained using the full-length proteins. For the ten homologous SAP proteins identified, we conducted a systematic analysis using various bioinformatic tools. The results showed that they all possessed molecular features similar to those of CcSAPs, such as an SG-rich domain in the N-terminal portion, a transmembrane domain that shared relatively high similarity (only within species), a conserved polypeptide segment after the transmembrane domain (only within species), and multiple phosphorylation sites (Table 2). These observations suggested that SAPs among different diatom species did not share significant sequence homology but possessed conserved motifs, the same molecular features, and amino acid composition, just like other biosilica-associated proteins (*e.g*., silaffins and cingulins) [25, 50].

**Table 2:** Basic characters of different regions of *SAP*s homologues

Species	Protein ID	% Ident	E-value	N-terminal			C-terminal			TM		
				pI	SG (%)	PS	pI	SG (%)	PS	pI	SG (%)	PS
*T. pseudonana*	25736	65	1.07E−33	5.31	32.8	28	5.15	14.8	5	5.52	8.7	1
*T. pseudonana*	11967	55.2	4.46E−33	4.43	28.9	37	9.4	16	7	5.19	17.3	0
*T. pseudonana*	25805	50	1.01E−53	10	43.6	44	10.25	20.3	4	5.52	15.8	3
*F. cylindrus*	229008	29.3	8.63E−06	4.43	62.3	34	10.77	11.3	0	5.57	5.3	7
*F. cylindrus*	249181	29.3	1.79E−06	5.85	61.7	34	10.77	11.3	0	5.57	10.6	7
*F. cylindrus*	277621	31.5	2.45E−06	8.1	69.4	25	10.77	11.3	7	5.57	10.6	0
*M. variabilis*	796099	52	1.18E−16	7.95	30.9	6	9.86	21.1	0	5.52	13	5
*M. variabilis*	717569	44.2	6.80E−08	5.52	34.6	47	4.56	7	16	5.52	14.3	0
*T. oceanica*	71447	51.2	1.58E−17	7.8	45.2	25	8.76	16.7	6	5.52	13.6	0
*T. oceanica*	81794	34.8	5.79E−06	4.01	40.8	32	6.85	25.4	24	5.52	13	0

pI, isoelectric point; PS, putative number of phosphorylation sites; TM, transmembrane region; SG indicate the total content of serine and glycine

### Transcriptional characteristics of *CcSAPs*

Tesson et al. reported that *TpSAP1* and *TpSAP3* were closely linked to the frustule phenotypes in *T. pseudonana*. However, knockdown of these two genes only affected the phenotype of the distal valve surface, such as the silica network pattern and extent of silica deposition, which has little or no impact on hemostasis [29]. Previous studies have suggested that a porous basal layer, formed during the very early stages of frustule biogenesis, defines the area of silica deposition and the morphological features of the frustule [50–52]. Therefore, we aimed to identify the *CcSAPs* that function in the early stages of biosilica morphogenesis, as genetic manipulation of these genes is more likely to cause significant alterations in frustule morphology.

Same as in other diatom species [45, 53], cell cycle progression in *C. cryptica* is dependent on silicate availability. Deprivation of silicate leads to the arrest of *Cyclotella* cells at the G2 stage, during which the new frustule begins to form, and biosilification genes are highly transcribed. Upon silicon replenishment, the frustule formation process of the majority of *Cyclotella* cells can move forward synchronously. Around 1–2 h later, cells enter G1 phase; and after around eight hours, cells enter the second G2 stage. Therefore, transcript patterns of different *CcSAPs* generated based on silicate starvation and replenishment synchronized cultures can provide valuable information for the identification of genes participating in the early stage of frustule biogenesis.

In this study, the transcript abundance of three *CcSAPs* under seven continuous timepoints (the first four and later three were silicate starvation and replenishment, respectively) were investigated, and the results demonstrated that three *CcSAPs* displayed three transcriptional patterns (Additional file 1: Fig. S3). For *CcSAP1*, the transcriptional abundances remained stable across the whole time. During silicate starvation period, both *CcSAP2* and *CcSAP3* were up-regulated and peaked at t = 0 h (silicate starvation for 48 h). After silicate replenishment, their transcriptional abundances began to decrease drastically. Until t = 8 h, *CcSAP3* still displayed a decrease trend, whereas *CcSAP2* began to rise again. Such observations suggested that *CcSAP2* likely plays an important role in the early stage of frustule biogenesis, whereas *CcSAP3* may participate in the late process. Therefore, *CcSAP2* was chosen for further investigation in this study.

### Screening of transgenic* C. cryptica*

**Fig. 1:**
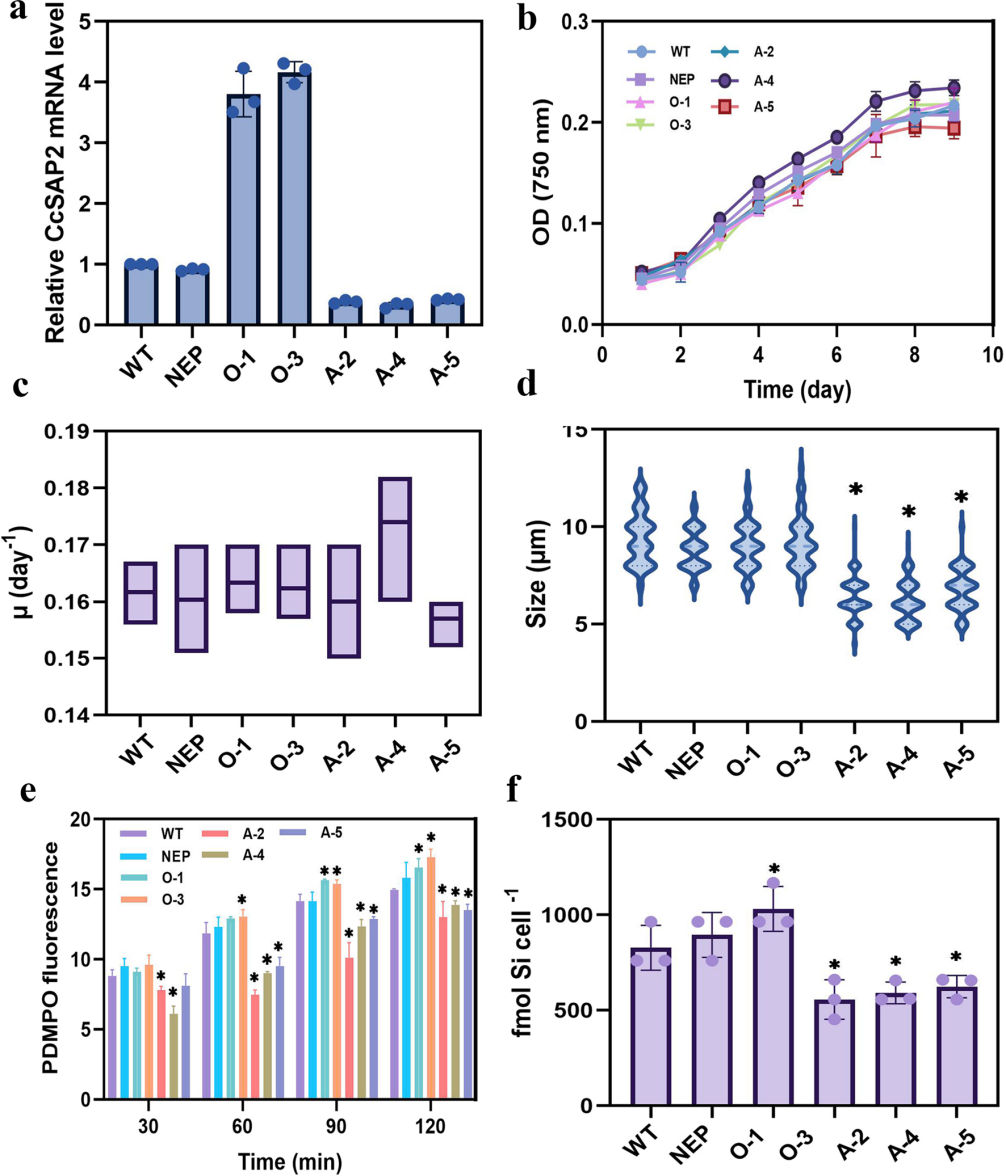
Validation of *CcSAP2* manipulation strains. The *CcSAP2* transcriptional abundances (**a**), growth curves (**b**), specific growth rates (**c**), cell sizes (**d**), PDMPO fluorescence intensities (**e**), and biosilica contents (**f**) of different *C*. *cryptica* lines. NEP, *C*. *cryptica* transformed with empty CcpPHa-T1 vector; O-1 and O-3, *CcSAP2* overexpression strains; A-2, A-4, and A-5, *CcSAP2* knockdown lines; fluorescence intensity was detected within 2 h after silicate replenishment; * indicates significant difference from WT (*p* < 0.05)

The reconstructed vector of CcpPha-T1 was adopted for both overexpression and antisense RNA knockdown to identify the role of *CcSAP2* on biosilica formation. After being screened by f/2 solid medium containing 0.1 μg mL^–1^ Zeocin, primers corresponding to *ble* gene (Ble-fw *and* Ble-rv) were used to detect the successful transformation based on genomic DNA. All ten transformed strains (five overexpression and five knockdown) displayed bands with expected length, but not the WT strain (Additional file 1: Fig. S4), indicating successful integration of the plasmid into the genome. Two overexpression strains (designated as O-1 and O-3) and three antisense RNA knockdown strains (designated as A-2, A-4, and A-5) were randomly selected for further transcriptional analysis. qRT-PCR results showed that the transcriptional abundances of *CcSAP2* in the overexpression strains were approximately four times higher than the WT and NEP strains, while that of the knockdown strains was only around 40% of the WT strain (Fig. 1a). As biosilica-related proteins undergo complex post-translational modifications [29, 54], it is challenging to obtain specific antibodies for protein quantification analysis. Moreover, both the N-terminal and C-terminal of *CcSAPs* possess several cleavage sites, making it difficult to conduct quantitation analysis using an anti-His tag antibody [29]. Thus, we did not perform translation verification in this study.

### Physiological properties of *CcSAP2* transformants

Both transformants and WT reached their maximum cell concentrations (0.21–0.25 of OD_750_) at Day 10 and showed growth performances similar to each other (Fig. 1b). Additionally, same situation was also observed from the specific growth rates (Fig. 1c). Though the specific growth rate and final cell density of A-4 and A-5 strains were different with the control groups, no significant differences were obtained among them, suggesting that the genetic manipulations targeting to *CcSAP2* did not result in significant influence on the growth performance of *C. cryptica.* The cell size of the *CcSAP2* overexpression lines remained similar to that of the WT and NEP strains. However, the cell size of antisense clones (A-2, 4, 5) varied only from 6.2 μm to 7.0 μm, approximately 28–34% lower than that of the WT strain (9.2 ± 1.19 μm) (Fig. 1d).

To further evaluate the influence of *CcSAP2* on silica biogenesis, the silica incorporation dynamics of different strains were compared by staining newly formed biosilica with PDMPO. In this method, the amount of silica incorporated into the cell wall was represented by fluorescence intensity. After 48 h of silicon starvation, sodium silicate with a final concentration of 25 µM was added to the *C. cryptica* cultures to initiate cell wall synthesis. As shown in Fig. 1e, the silicate incorporation contents of the three knockdown mutants were significantly lower than those of the WT and NEP strains at nearly all four time points. Interestingly, although the overexpression lines exhibited similar cell size to the WT, their PDMPO intensities were higher than those of the WT. This observation also coincided with our cellular biosilica results, which showed that the cellular silica contents of *CcSAP2* knockdown and overexpression lines were lower (18–25%) and higher (7–24.5%) than those of the WT strain (Fig. 1f). Both of these results confirmed that *CcSAP2* might play important roles in both frustule shape formation and silica deposition.

### Morphological characters of frustules from different *C. cryptica* lines

**Fig. 2:**
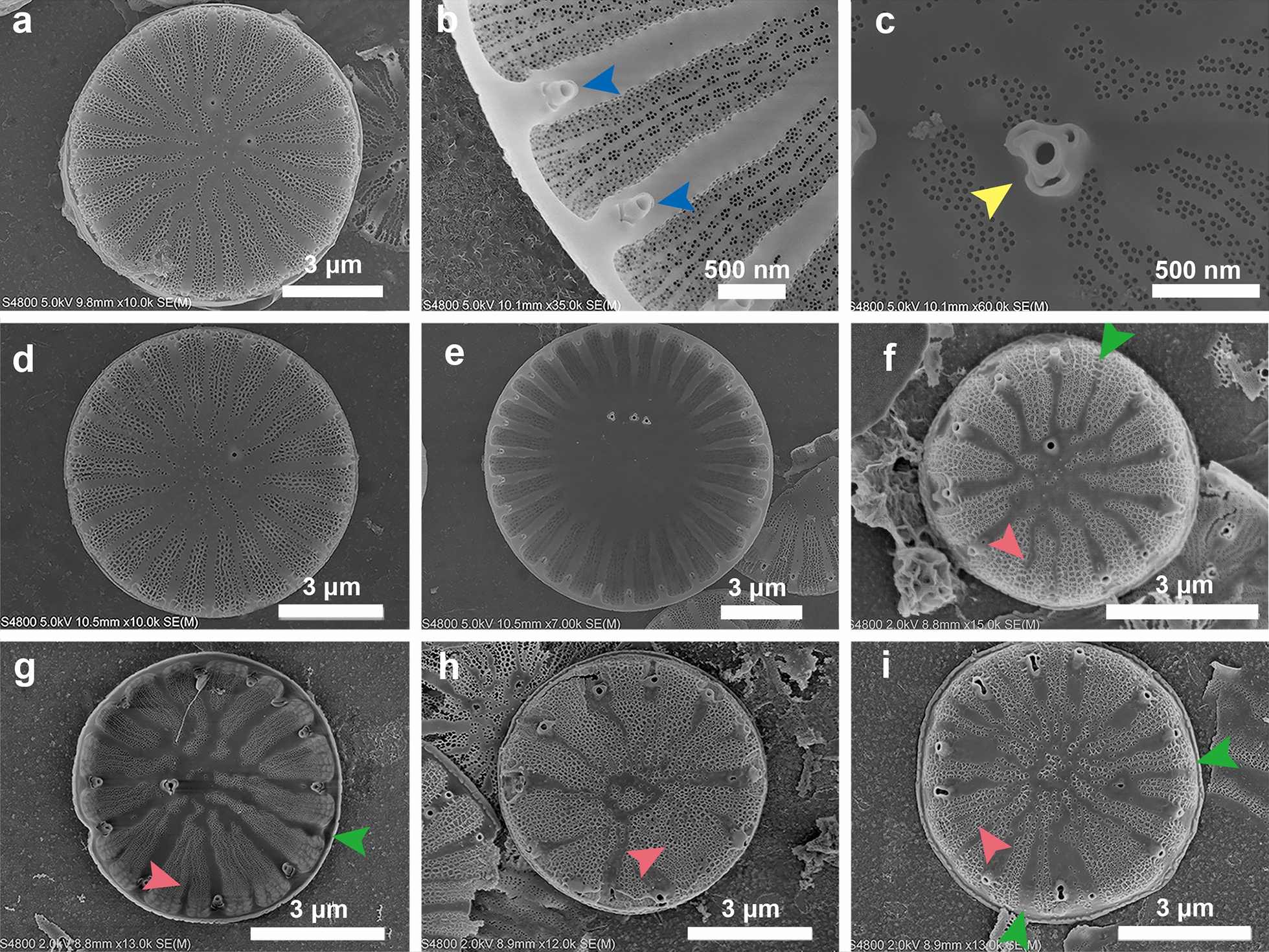
SEM images of different *C*. *cryptica* lines. WT, overexpression, and knockdown lines are shown in **a**–**i**. Blue and yellow arrows indicate fultoportulae on the valve rim and fultoportula on the central area. Green and pink arrows point out the missed fultoportulae and abnormal ribs

The frustule of *C. cryptica* is cylindrical and consists of two circular valves, the top epitheca and bottom hypotheca, which are connected by multiple overlapping girdle bands. The valve has a diameter of around 10 μm, and its distal surface (external side) is characterized by alveolate striae and a central area (as shown in Fig. 2). The striae are arranged in a radial pattern and filled with nanoscale pores. The central area, which spans around 1/3 to 2/3 of the valve surface, is relatively smooth compared to other areas and sporadically ornamented with granules. Multiple fultoportulae (tube-like structures) are regularly distributed on the valve rim and between the striae in most cases (blue arrows in Fig. 2b). One or two (rarely three) fultoportulae, which are relatively flat compared to the fultoportulae on the valve rim but have a similar shape, are located on the central area and can be observed from both the distal surface and the proximal surface (interior side) (yellow arrow in Fig. 2c).

The morphology of the valves of the overexpression strains showed no visible differences from the WT strain in this study (Fig. 2d, e). However, the microstructures of the knockdown strains were quite distinct (Fig. 2f–i). Similar to the results observed under the light microscope, the SEM images clearly demonstrated a much smaller valve diameter of the knockdown lines than the WT strain. Moreover, the originally flat central area shrank largely in the knockdown lines, and multiple nanoscale pores appeared on it (Fig. 2f–i). Obvious discrepancies could also be observed in the striae. For the WT strain, the width and line-type of different striae on the same valve were basically the same, and the boundary between two adjacent striae was clear. In comparison, the knockdown lines exhibited much wider striae with an irregular distribution pattern, and the boundary between striae became ambiguous or even disappeared in some cases (pink arrows) (Fig. 2f–i). In the WT strain, the fultoportulae, having the same number as the striae, distributed accurately on the valve rim. However, some fultoportulae in knockdown lines disappeared (green arrows), and their localization transferred from the valve edge to the valve surface in some cases.

### Physicochemical properties of *CcSAP2* transformants

The silica content and morphological characters are crucial factors underlying the physicochemical properties of the frustule. The reduction in cell size, shrinking central area, decreased striae boundary, and widening striae can potentially increase the BET surface area, porosity, and absorption ability, which can further enhance the hemostatic efficiency. Therefore, the above observations prompted us to investigate the physicochemical properties of *CcSAP2* transformants. Moreover, since the morphological characters of the overexpression strains did not show any significant alterations compared to the WT strain, only the knockdown lines were tested in the subsequent experiments.

The N_2_ adsorption desorption isotherm (Additional file 1: Fig. S5) demonstrated, as expected, that the frustules of all three knockdown strains displayed significantly higher BET and pore volume than the WT (Table 3). For example, A-2 reached a BET value of 116.5 m^2^ g^–1^, 1.8 times higher than the WT. The pore volumes of the three knockdown strains varied within the range of 0.2–0.25 cm^3^ g^–1^, 1.1–1.5 times higher than the WT. In contrast to the BET and pore volume, knockdown of *CcSAP2* resulted in a decrease in pore diameter. Among the three knockdown lines, A-2 achieved the lowest pore diameter of 8.97 nm, which was around 22% lower than the WT.

**Table 3:** The physical characteristics of four frustules

Strains	BET (m^2^ g^–1^)	Pore volume(cm^3^ g^–1^)	Pore diameter (nm)
WT	66.5 ± 1.06	0.1704 ± 0.01	11.56 ± 1.38
A-2	116.5 ± 6.82*	0.2537 ± 0.01*	8.97 ± 0.76*
A-4	97.1 ± 2.25*	0.1992 ± 0.01*	10.79 ± 2.32
A-5	86.7 ± 1.11*	0.2407 ± 0.01*	10.42 ± 1.54*

^*^ indicates the significant difference between WT and mutants; *p* < 0.05

**Fig. 3:**
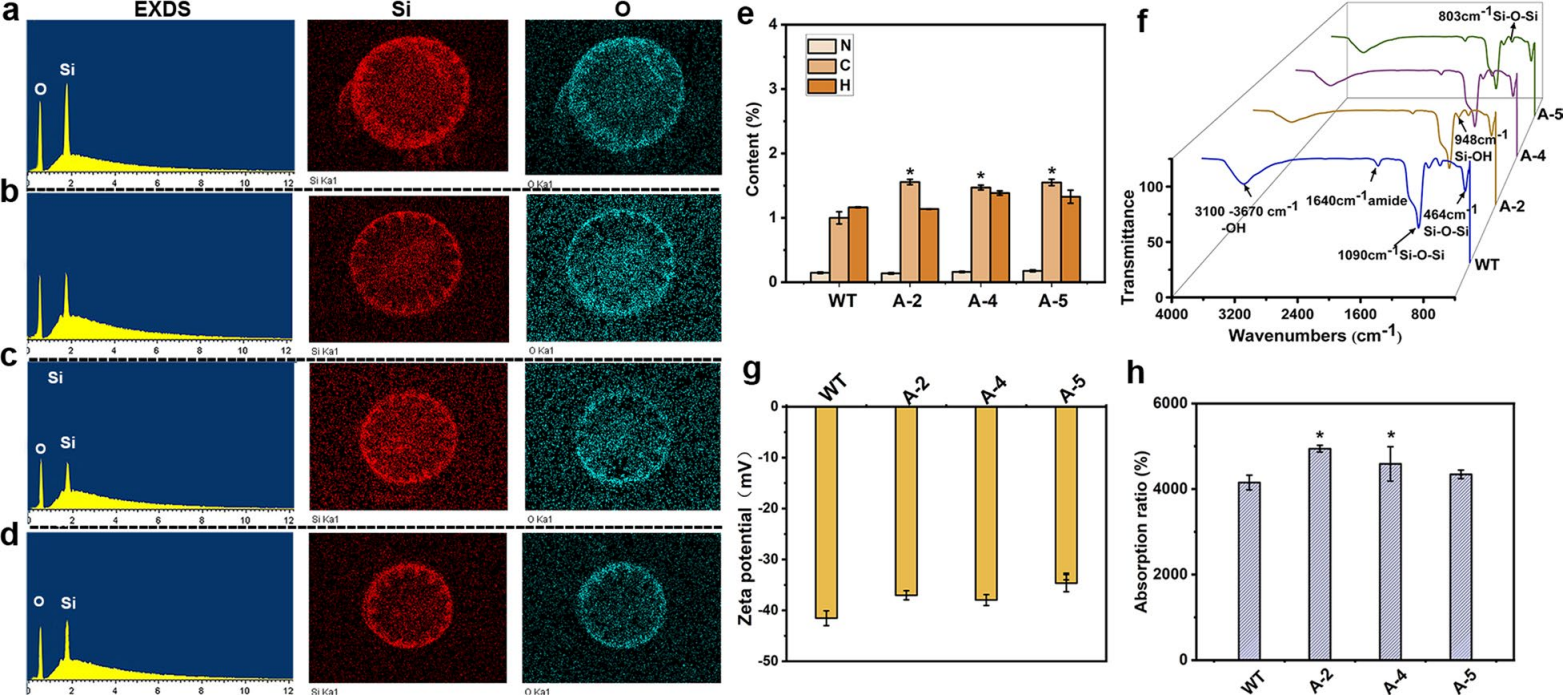
Characterization of diatom frustules. EDXS analysis of WT, A-5, A-4, and A-2 are shown in **a**–**d**; element content, FTIR spectra, and zeta potential of four *C*. *cryptica* lines are shown in e–g. A-2, A-4, and A-5 represent the antisense RNA clones; * indicates the significant difference from WT (*p* < 0.05)

The EDXS results demonstrated that frustules of both the WT and knockdown strains were mainly composed of silicon and oxygen (Fig. 3a–d). Element analysis was further conducted to reveal whether the chemical composition of different *C. cryptica* lines was different. The results indicated that the nitrogen and hydrogen contents among different strains were similar to each other, whereas the carbon contents of the knockdown strains (varying from 1.47–1.56%) were higher than the WT (1%) (Fig. 3e). As for the FTIR analysis, both the WT and knockdown spectra were featured by siloxane bonds (Si–O-Si, peaks at 1090, 803, and 464 cm^–1^) and silanol groups (Si–OH, peaks at 948 cm^−1^). Moreover, amide groups (C = O, peak around 1645 cm^–1^) were also observed, suggesting that trace amounts of organic substances still retained in the frustules (Fig. 3f). Zeta potential exhibited that all diatom frustules possessed a negative charge (varying from – 35 mV to – 41 mV), and the WT was more highly charged than the knockdown strains (Fig. 3g). As for absorbability, all knockdown strains showed a higher liquid absorption ratio than the WT, and the highest value was obtained from A-2 (approximately 49 times the weight of liquid), around 19% higher than that of the WT (41 times the weight of liquid) (Fig. 3h).

In the knockdown lines, BET, pore diameter, and liquid absorption capacity, all of which have been proposed as pivotal factors underlying the hemostasis properties of diatoms [11], diverged great from that of the WT strain, driving us to conduct a comparative analysis about the hemostatic performance between WT and knockdown lines further.

### In vitro coagulation evaluation

#### Blood clotting time and thromboelastogram analysis

**Fig. 4:**
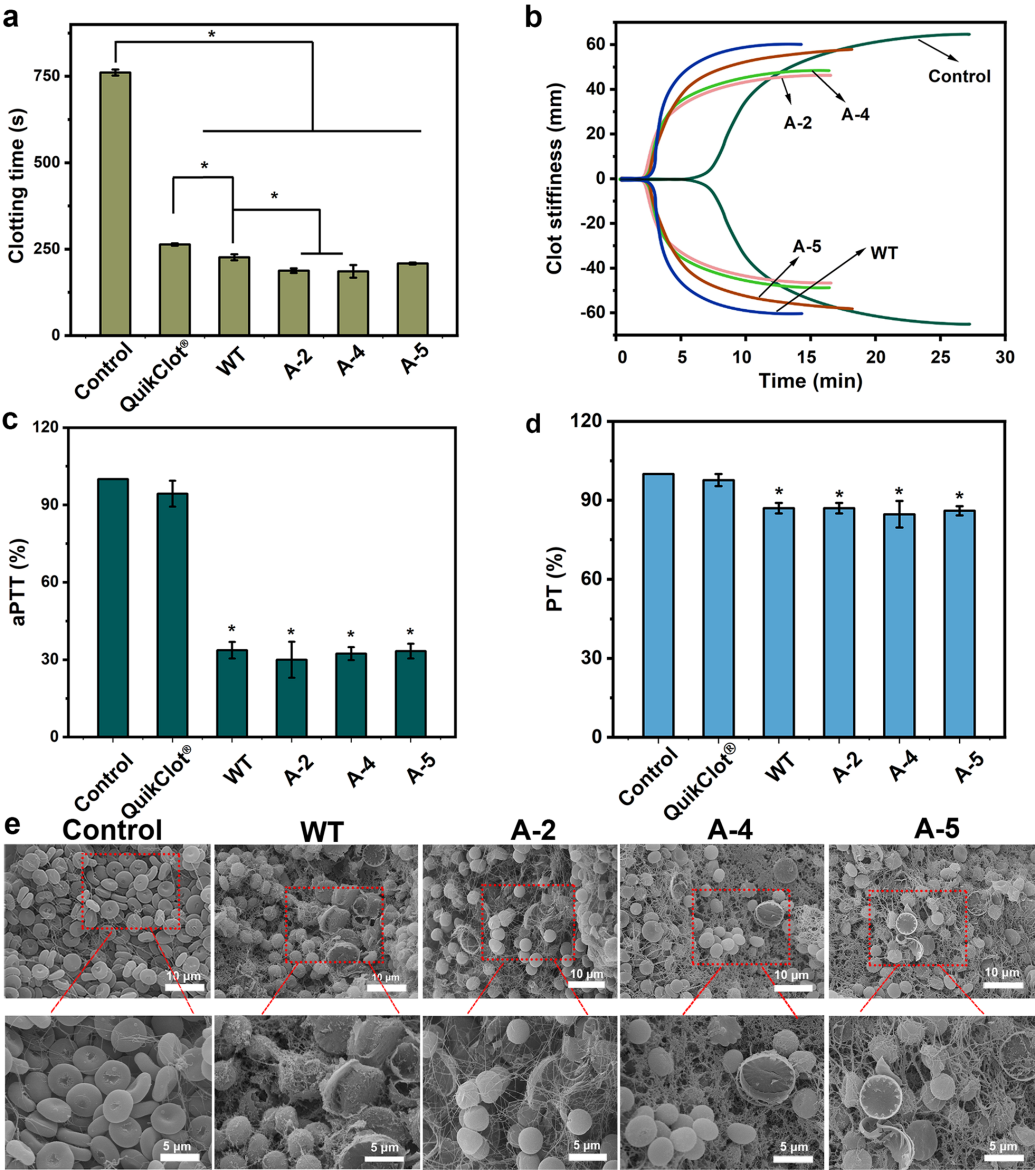
In vitro blood coagulation performance of frustules. **a**–**d** The in vitro blood clotting time, TEG curve, aPTT, and PT of different treated groups. **e** The SEM images reflecting the interaction between frustule and red blood cell. *in a represents the significant difference (*p* < 0.05); * in **c** and **d** represents the significant difference from QuikClot^®^ (*p* < 0.05)

The coagulation properties of different materials were evaluated based on in vitro blood clotting time, and all frustule-treated groups showed much shorter clotting time than QuikClot^®^ and blank control (Fig. 4a). As for different frustules, the clotting time of three knockdown strains, ranging from 135 s (A-2) to 173 s (A-5), was shorter than that of the WT (187 s), and no significant difference could be obtained between A-2 and A-4.

TEG analysis was conducted to further explore the discrepancies associated with the entire coagulation process. The values of four clotting-related parameters (R, K, angle α, and MA) are shown in Fig. 4b and Table 4. The R and K values indicate the time for initial clot formation and clot enlargement to 20 mm. The alpha angle represents the angle between the baseline and the tangent curve to the K value and can be used to evaluate the speed of clot formation. The MA value indicates the width of the widest gap in TEG tracing and can be used to reflect the maximum strength of the clot. As for these four parameters, frustules achieved much shorter R and K values as well as much higher angle α than the blank control group (Fig. 4b and Table 4). For example, the R and K values for the blank control were 9 and 3.3 min, while the frustules exhibited values within the ranges of 3.3–3.8 min and 1.2–1.3 min, only around 1/3 of the blank control group. Such an observation demonstrated that frustules can largely shorten the formation time of the initial clot and accelerate the process of blood coagulation. Regarding different frustules, though all four frustules exhibited close K values, significant differences were observed in the other three parameters between the knockdown and WT strains. For example, A-2 and A-4 showed R values of 3.3 and 3.5 min, both of which were significantly lower than the WT (Fig. 4b and Table 4). Moreover, the angle α values of A-2 and A-4 (69.7° and 66.5°) were significantly higher than the WT (62.8°). All of the above suggests that the knockdown strains can initiate blood coagulation and form a clot at a higher speed than the WT. Besides that, all *C. cryptica* frustule groups showed lower MA values than the blank control group, but still within the normal range (50–70 mm) [55, 56] (Fig. 4b and Table 4).

**Table 4:** TEG parameters of four *C. cryptica* lines

	R (min)	K (min)	Angle (°)	MA (mm)
Blank control	8.96 ± 1.23	3.3 ± 0.1	47.72 ± 3.01	71.87 ± 0.99
WT	3.76 ± 0.15^#^	1.17 ± 0.05^#^	63.2 ± 3.62^#^	63.6 ± 2.08^#^
A-2	3.16 ± 0.32^*#^	1.26 ± 0.05^#^	70.13 ± 6.86^*#^	54.8 ± 1.05^*#^
A-4	3.43 ± 0.05^*#^	1.3 ± 0^#^	68.33 ± 3.97^*#^	54.56 ± 1.91^*#^
A-5	3.67 ± 0.15^#^	1.3 ± 0.2^#^	66.93 ± 1.88^*#^	64.73 ± 4.05^#^

^#^ and ^*^ mean the significant difference (*p* < 0.05) with blank control group and WT, respectively

In summary, compared to the WT strain, the genetic modified frustules, with larger specific surface area and pore volume as well as smaller particle size and pore size, can accelerate the initiation of coagulation cascade system due to their strong ability to concentrate coagulation factors and platelets.

#### Plasma clotting analysis

To confirm the coagulation pathway, PT and aPTT tests were performed. As shown in Fig. 4c, the aPTT values obtained from diatom groups were significantly lower than two control groups (accounting for 32–34% and 32–35% of the blank control and QuikClot^®^ control, respectively), meaning that frustules can induce the intrinsic coagulation pathway. As for the PT, though the frustules values were lower than two control groups (blank and QuikClot^®^), no significant difference was obtained from them (Fig. 4d). SEM images reflected the status of blood clot. Abundant erythrocytes were found to be aggregated around frustules, and both erythrocytes and frustules were wrapped by large amounts of fibrin strictly (Fig. 4e).

### In vivo hemostasis assay

#### Rat tail amputation

**Fig. 5:**
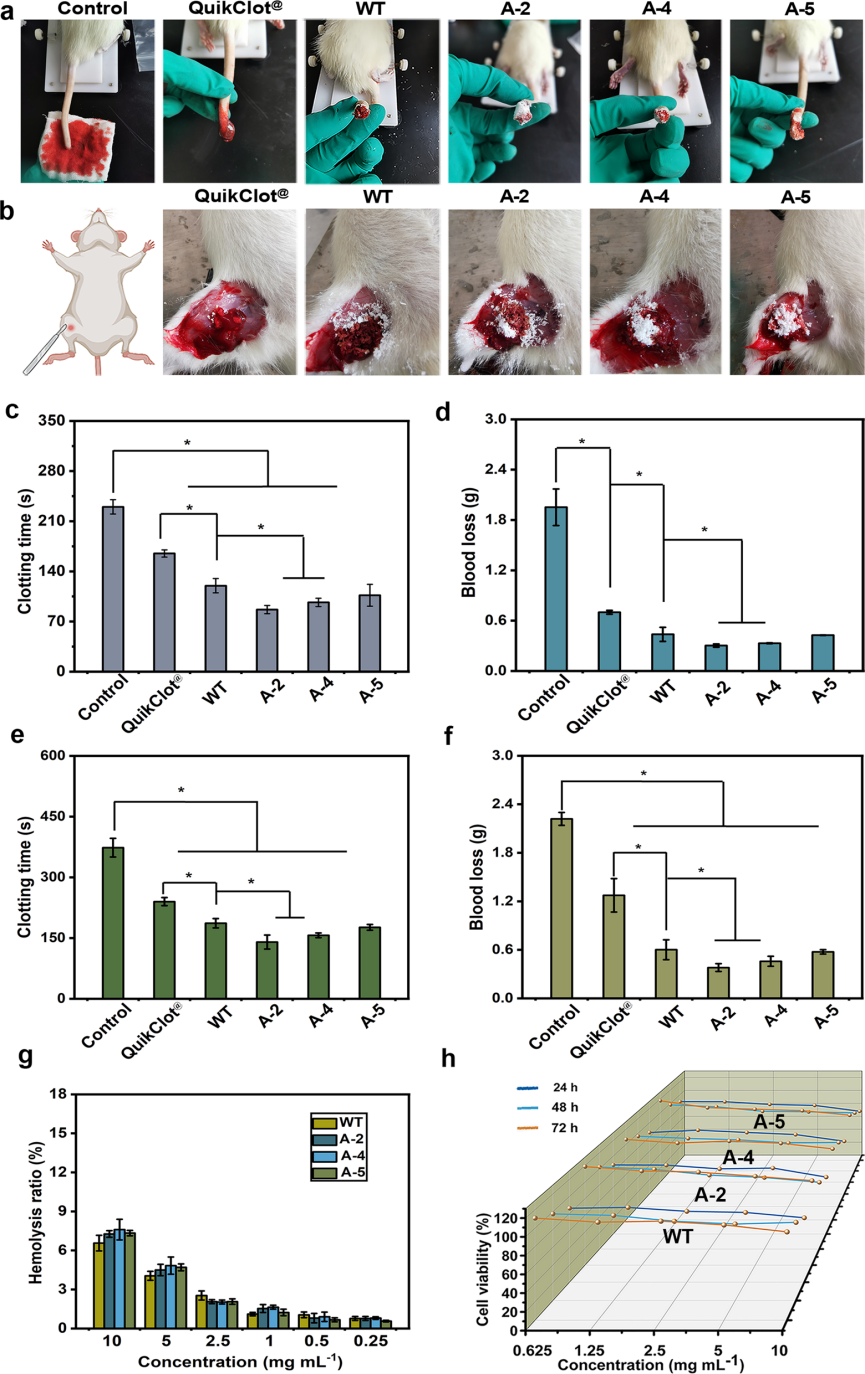
In vivo hemostasis performance of frustules. **a** and **b** Represent the rat tail amputation and femoral artery model; **c** and **d** mean the hemostatic time and blood loss weight in the rat tail model; **e** and **f** are the hemostatic time and blood loss weight in the femoral artery model; **g** means the blood compatibility of frustules; **h** represents the cell compatibility of frustules at 24 h, 48 h, and 72 h. * represents the significant difference (*p* < 0.05)

Rat tail injury and rat femoral artery models were adopted to comparably evaluate in vivo hemostatic effect of different types of frustules (Fig. 5a). In the former model, QuikClot^®^ showed a hemostasis time and blood loss of 165 ± 5 s and 0.69 ± 0.02 g, whereas that of *C*. *cryptica* frustules largely reduced these two values to the ranges of 86.7–120 s and 0.3–0.44 g (Fig. 5c–d). Furthermore, significant difference in hemostatic efficiency also could be observed within four types of frustules. For example, the shortest clotting time was obtained from A-2 (86.6 ± 5.8 s), 27.5% and 48% shorter than that of the WT (120 ± 10 s) and QuikClot^®^ (165 ± 5 s). A-4 achieved a hemostasis time of 96 ± 5.7 s, also significantly shorter than the WT. The clotting time of A-5 was also shorter than WT but showed no significant difference. The situation of blood loss was similar to the clotting time, both A-2 (0.3 ± 0.02 g) and A-4 (0.33 ± 0.01 g) were significantly lower than the WT (0.44 ± 0.08 g).

### Rat femoral artery model

The results of rat femoral artery model further confirmed that the *CcSAP2* knockdown strains can significantly shorten the hemostasis time and reduce the blood loss. Same with the results observed from rat femoral artery model, A-2 achieved the hemorrhage control within the shortest time of 140 ± 17 s, 42% and 25% shorter than the QuikClot^®^ and WT strain (Fig. 5b and e). The knockdown strains of A-4 (156 ± 5 s) and A-5 (176 ± 7 s) ranked the second and the third, both of which were also largely shorter than QuikClot^®^. Moreover, *CcSAP2* knockdown strains also exhibited much lower blood loss compared to the blank control group and WT strain (Fig. 5f). Particularly for A-2, the blood loss value (0.38 ± 0.04 g) only accounted for 30% and 63% of the QuikClot^®^ and the WT strain, respectively.

### Biocompatibility evaluation

The biocompatibility of frustules was evaluated via in vitro hemolysis and cytocompatibility, and 0.9% NaCl and MilliQ water were taken as negative and positive controls. As shown in Fig. 5g, the hemolysis ratio of diatoms was highly concentration dependent. The highest ratio of 7.6% was displayed by A-4 frustule at a concentration of 10 mg mL^−1^. When the frustule concentration decreased to 5 mg mL^–1^, the value drastically reduced to 4.8%. Under concentrations below 5 mg mL^−1^, frustules showed favorable blood compatibility (Fig. 5g and Additional file 1: Fig. S6). In terms of cytotoxicity, our results demonstrated that the cell viability at all tested concentrations remained at a high level (> 80%) after incubation for 24 h (Fig. 5h). The cell viability of L929 cells increased to 94–118% when the incubation time was extended to 72 h (Fig. 5h). All above results illustrated that frustules can be utilized as a safe biomaterial for hemorrhage control.

### Correlation analysis between physicochemical properties and hemostasis efficiency

**Fig. 6:**
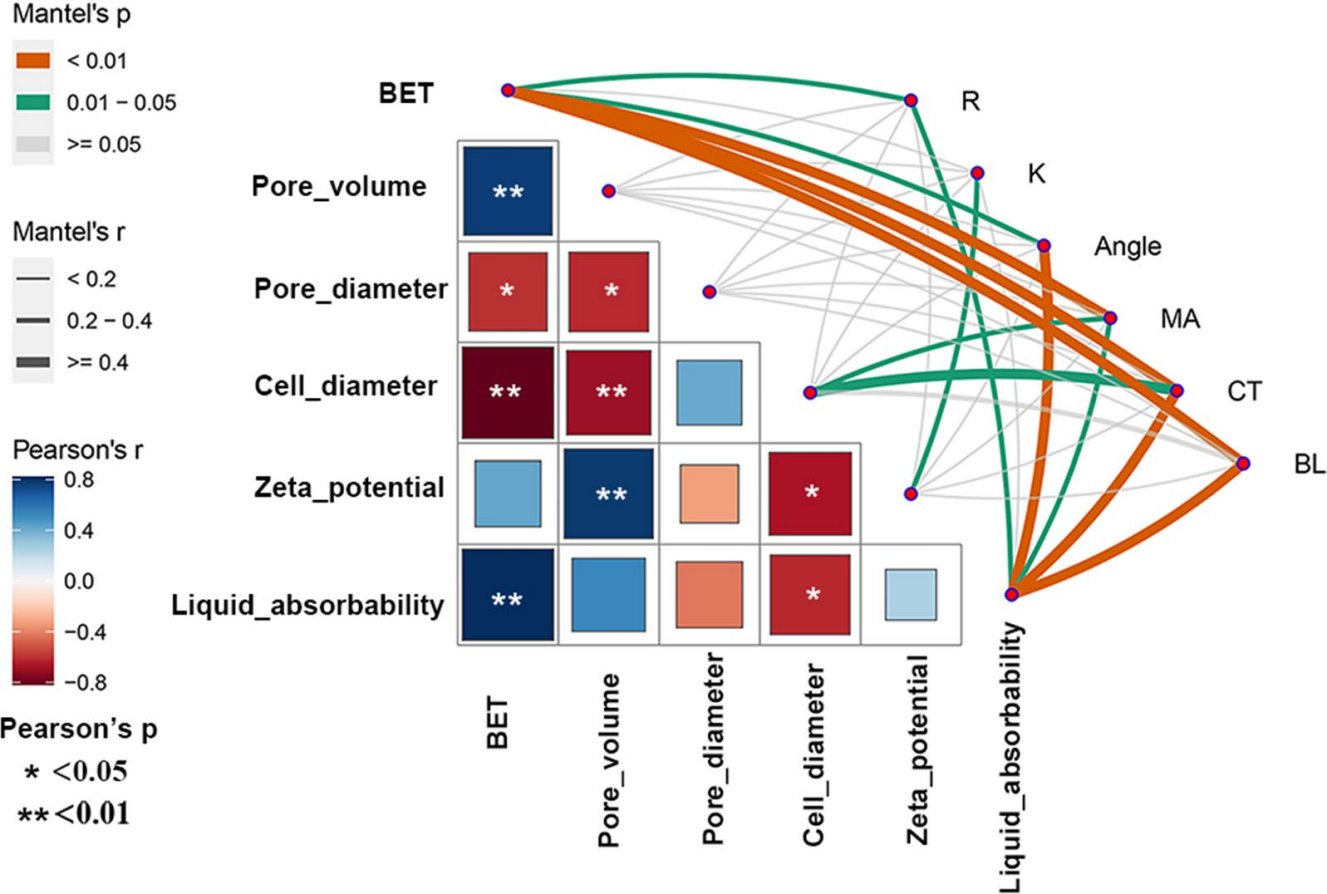
The Mantel test results of morphology characteristics and coagulation effect of diatom frustules

The Mantel test results demonstrated that BET and liquid absorbability were the most essential parameters associated with hemostasis performance (Fig. 6). For example, BET was found to be strongly correlated (r > 0.5) with MA, in vivo clotting time, and blood loss weight (*p* < 0.01); and moderately correlated (0.3 < r < 0.5) with R and angle α (*p* < 0.05). Similar situations could also be obtained from the liquid absorbability, which was strongly correlated with in vivo clotting time and blood loss weight (*p* < 0.01); and moderately correlated with R (*p* < 0.05) and angle α (*p* < 0.01). These results suggested that frustules with high BET and large liquid absorbability can largely shorten the formation time of initial clot and accelerate the coagulation speed, both of which will further contribute to the shortening of in vivo clotting time and the reduction of blood loss weight. Zeta potential was found to be moderately correlated (0.3 < r < 0.5) with K (*p* < 0.05), demonstrating its key role in clot enlargement. Additionally, a strong correlation (r > 0.5) between BET and MA was also obtained, suggesting that frustules with quite high BET may weaken the strength of the clot. Furthermore, a low correlation (r < 0.3, *p* < 0.05) was obtained between liquid absorbability and MA, which implies that extraordinarily high absorbability may also have a negative effect on clot strength. Taking into consideration the BET and liquid absorbability values (Table 2) and the MA values (Table 3), it can be further calculated that either a BET value higher than 122.82 m^2^ g^−1^ or a liquid absorbability value higher than 50.35 times its own weight of liquid will generate an MA value lower than 50 mm (Additional file 1: Fig. S7). Additionally, cell diameter also displayed moderate correlation with MA and clotting time. However, Pearson's analysis demonstrated that cell diameter was highly correlated with both BET and liquid absorbability, and thus we did not consider it as an independent parameter associated with hemostasis performance here.

## Discussion

The excessive reduction of total amount blood will cause serious consequences, *e.g*., cerebral hypoxia, hemorrhagic shock, and even death [57, 58]. The amorphous silica composition, multilayer porous valve, internal hollow structure, and abundant functional groups on the surface endow diatom frustules with huge potential for hemostatic uses [59, 60]. In this study, both the WT and knockdown strains of *C*. *cryptica* biosilica demonstrated significantly shorter clotting time and less blood loss weight than the QuikClot^®^. For example, in rat tail amputation model and femoral artery model, the clotting time of QuikClot^®^ were 165 s and 186 s, while that of A-2 frustule were 86 s and 140 s. Taking low levels of hemolysis, good cytocompatibility, and easy availability into consideration together, diatom frustule can be regarded as a promising biomaterial for rapid bleeding control [10].

### Target genes for the adjustment of hemostatic performance by genetic manipulation strategy

Previous reports indicated that the morphological characters of the frustule, such as valve diameter, pore size, and pore density, maybe the key factors determining their hemostatic efficiency [10, 11]. Therefore, it is of significance to establish a feasible strategy to regulate the morphological characters of frustule, by which the hemostatic performance can be further enhanced. Compared to other methods, such as HF etching and cultivation condition adjustment, genetic manipulation shows various advantages in frustule morphology modification [61]. However, the frustule morphogenesis mechanism is still unclear, which hinders the fulfillment of this strategy.

It has been recognized that (1) silicalemma-spanning proteins play important roles on frustule formation by interaction with cytoskeleton or other cellular components; (2) the frustule morphogenesis occurs in distinct steps. The initial stage is related to the formation of base layer, defining the basic structure (*e.g.*, cell diameter, striae distribution pattern, and central area localization) in x, y-axes; polymerization across the whole base layer in z-axis occurs in the later stage, determining the microstructures of valve (*e.g.*, thickness, costae, and areola pore) [51]. Based on this knowledge, it can be inferred that SDV transmembrane proteins, which function in the early stages of valve formation, are desirable targets for frustule morphology modification. Several molecular features have indicated that SAPs are SDV transmembrane proteins associated with frustule biogenesis. However, knocking down *TpSAP1* and *TpSAP3* only resulted in the alteration of the distal surface microstructure of *T. pseudonana* [29]. Herein, the transcription of *CcSAP2* under silicate replenishment condition up-regulated much earlier than *CcSAP1* and *CcSAP3*, implying that this gene maybe relevant to the formation of base layer. Therefore, *CcSAP2* was identified as the target gene in this study. In contrast to the moderate variation caused by the knockdown of *TpSAP1* or *TpSAP3*, the knockdown of *CcSAP2* resulted in significant alterations to frustule morphology. This observation reinforces previous speculations about the role of SDV transmembrane proteins in frustule morphogenesis. On the other hand, it also suggests that *SAP2* functions differently from *SAP1* and *SAP3*.

The alteration of morphologic characters of the frustule further imposes great influences on their hemostatic performance. Both in vitro and in vivo experiments demonstrate that the frustules of knockdown lines display shorter clotting times, lower blood loss, and faster blood coagulation speeds compared to the WT line. Such enhancements can be attributed to the improved BET, pore volume, and liquid absorption ratio of the knockdown lines. All these results provide evidence that genetic manipulation based on genes encoding silicalemma-spanning proteins is a viable strategy for regulating frustule morphology and enhancing the hemostatic properties of frustules. Therefore, it deserves more attention to explore other silicalemma-spanning proteins, particularly those functioning in the early stages of biosilica biogenesis, in the future. Additionally, different with previous reports focusing on surface modification or immobilization [13], this study explored a novel strategy to enhance the hemolytic activity of diatom biosilica. Actually, to the best of our knowledge, this is the first report of enhancing the hemostasis effect of frustule based on genetic manipulation method and can open the way to a new series of bioengineered frustules with desired microstructures for hemostasis agents uses.

### Key physicochemical properties underlying the hemostasis efficiency

By fabricating synthetic mesoporous silica with specific gradient properties, it becomes possible to identify the key property determining hemolytic activity and also aids in clarifying the hemostasis mechanism. For instance, using mesoporous silica nanoparticles with various sizes (ranging from 60 to 220 nm) and pore diameters (5, 10, and 15 nm), Chen et al. found that nanoparticles with a pore diameter of 15 nm exhibited much shorter clotting times compared to those with 5 nm and 10 nm pores, while variations in particle size had little impact on coagulation [5]. Utilizing silica nanoparticles with different diameters (ranging within 4–85 nm), Kushida et al. demonstrated that the intrinsic coagulation activity of silica was size-dependent: small particles with high surface curvature were coagulation-inert, whereas larger particles with low surface curvature exhibited denaturation and subsequent coagulation [62]. Yoshida et al. examined the localization and biological response of silica nanoparticles with varying diameters (30, 70, and 100 nm) and found that the two smaller particles could activate the intrinsic coagulation cascade, suggesting that silica nanoparticles with a diameter of 100 nm or less may be safe for intranasal administration [63]. Such studies hold significant value as they can provide insights into the optimal direction for the rational fabrication of mesoporous silica materials.

When it comes to diatom frustules, conducting such studies poses challenges due to (1) species-specific frustule morphologies, with multiple morphological differences existing simultaneously among different species; and (2) the difficulty in quantifying many morphological features, especially fundamental ones such as frustule shape, pore shape, and pore arrangement patterns [10, 11]. Frustule mutants generated based on genetic manipulation provide a feasible strategy to overcome this obstacle. In this study, although the knockdown of *CcSAP2* resulted in alterations in cell size, pore diameter, pore volume, etc*.*, the fundamental characteristics of frustules remained similar among different lines, suggesting that that these frustules can be employed to identify key physicochemical properties associated with hemostasis efficiency. Bearing this in mind, Mantel test method was adopted to determine the correlation between physicochemical properties (*i.e.*, BET, frustule size, pore diameter, pore volume, liquid absorbability, and zeta potential) and hemostasis performances (*i.e*., R, K, Angle, and MA as well as in vivo clotting time and blood loss weight). Our results demonstrated that the high BET and large liquid absorbability promote initial clot formation and activation of the coagulation cascade, but exceeding certain boundaries can lead to the formation of clots with insufficient strength. Furthermore, a large negative zeta potential value is conducive to enhancing the rate of clot enlargement. These findings can provide valuable guidance for genetically manipulating frustule morphologies to achieve the desired hemostasis performance.

### The superiority of frustule as hemostasis agent

Capacities such as rapid bleeding control, good biocompatibility, and nontoxicity are only parts of essential requirements for hemostasis agent. An ideal hemostatic material should possess other additional effects beneficial for wound healing, *e.g.*, antibacterial infection, anti-inflammatory, the ability to facilitate angiogenesis, and the ability to promote the reconstruction of tissue functions [64]. Nowadays, though various materials including natural polymers, synthetic polymers, silicon-based materials, and metal-containing materials have been approved worldwide, few of them can meet all of the requirements listed above. Therefore, hybrid material, also known as composite material, which can integrate multiple useful properties of different materials and eliminate side effects of the single material, is attracting abundant attention [65].

Diatom frustules are outstanding substrates for hybrid material fabrication. Benefiting from rich silanol groups and siloxane bonds, the frustule surface can be easily modified through multiple approaches, including covalently bonded with drugs and antibodies, immobilized with hydrogel and polysaccharide, coated with metal ions and inorganic oxide layers [66]. Moreover, the internal hollow structure and porous surface also endow frustules with excellent loading capacity and sustained release property of drugs [67, 68]. All the above suggest that diatom frustules are suitable for fabrication as hybrid materials containing growth factors or specific drugs that can achieve rapid bleeding control and other beneficial effects for wound healing simultaneously.

Indeed, a distinct advantage of diatom frustules as hemostasis agents lies in their ability to be functionalized or modified by environment-friendly biosynthesis or biotechnology methods. In our previous study, calcium (coagulation factor IV) was successfully incorporated into frustules by adding calcium chloride to the cultivation medium of diatoms. Through this simple functionalization method, the hemolytic activity of frustules was significantly enhanced (the in vivo clotting time and blood loss weight of Ca-frustules only accounted for 1/2 and 1/3 of that of the original frustules) [12]. In addition to calcium, a variety of metal ions, many of which are also beneficial for rapid bleeding control (*e.g.*, aluminum and zinc), can be absorbed and incorporated into frustules by diatoms [69–71]. Another frustule functionalization strategy has been reported, although it has not yet been used in the hemostasis field. Using a specific biosilica protein (silaffin) as a localization tag, Kröger's team has developed a method called live diatom silica immobilization (LiDSI), through which functional enzymes and immunoglobulin G (IgG)-binding domains have been successfully immobilized onto the frustule surface [9, 72, 73]. Obviously, this enabling strategy can also be adopted to develop frustules as hybrid hemostasis agents. In addition to LiDSI, this study presented here is a case of frustule morphology modification through a biotechnology strategy. Considering the fact that only a limited number of diatoms can be efficiently cultivated on a large scale, this report provides a possible strategy for producing desired frustules based on one specific diatom species that can be easily cultivated.

As stated above, benefiting from unique physicochemical properties, easy availability, and environment-friendly enabling strategy, diatom frustule-based biosilica nanoparticles are an optimal option for rapid bleeding control and show huge potential for commercial application.

### Mechanism of frustule morphogenesis

Although the morphogenesis of diatom frustule has attracted abundant attention for a long time, it is still a huge challenge to explore biosilica-associated proteins and clarify the elusive relationship between specific gene and frustule phenotype, because only limited groups of organisms possess biosilification activity. In this study, the knockdown of *CcSAP2* led to huge alterations towards the frustule morphologies and significantly decrease of biosilica content, whereas the overexpression of *CcSAP2* only resulted in the increase of biosilica content without visible morphology changes. Such an unexpected discrepancy drove us to consider deeply about the mechanism of frustule biogenesis.

Nowadays, the facts that the formation of frustule occurs in SDV and the silicalemma-spanning proteins play essential roles on frustule biogenesis by interaction with cytoskeleton or other cellular components are definite [32, 51], while several important questions are still open for discussion. De Haan et al. discovered that the distal silicalemma is in very close proximity to the original plasma membrane, with a spacing distance of only 20 nm [32]. In fact, such a confined space is too narrow for microtubules to fit into, meaning that only the proteins located inside the proximal silicalemma can interact with the microtubules. However, this inference raises another problem: (1) all transmembrane proteins locate inside the proximal silicalemma, or (2) specific proteins span the proximal silicalemma, while others span the distal silicalemma and interact with unknown cellular components (or original plasma membrane) rather than microtubules. De Haan et al., also found that the mature silica valve is secreted outside by exocytosis, during which procedure the proximal silicalemma will fuse with the original plasma membrane generating the new plasma membrane, while the distal silicalemma will gradually disintegrate after exocytosis [32]. Other two studies conducted by Kotzsch et al. and Tesson et al. demonstrated that the C-terminal segments (cytoplasm domain) of silicalemma-spanning proteins will be cleaved off during biosilica formation [28, 29]. Taking all the above observations into consideration, it can be inferred that the C-terminal segments of proteins spanning the proximal silicalemma will experience different fates compared to those spanning the distal silicalemma. Specifically, the cleaved C-terminal segments of proteins spanning the proximal silicalemma are likely to remain in the cytoplasm, whereas those of proteins spanning the distal silicalemma will be (at least partially) secreted outside the cell. The intracellular localizations of *TpSAP1–3* have been investigated by Tesson et al., and the results demonstrated that the fluorescence signals of recombinant *TpSAP1* and *TpSAP3* were only present in forming girdle band and valves, but disappeared from the mature frustules. Meanwhile, the fluorescence signal of *TpSAP2* was observed within the cytosol [29]. Therefore, it is plausible to believe that the localization of SDV transmembrane proteins is protein type-dependent. If this is indeed the case, the different pI values observed in the transmembrane region in this study (Table 2) may be related to the localization of CcSAPs.

Another open question is about the role of the N-terminal domains of SAPs. Kotzsch et al. demonstrated that the silicalemma-spanning protein of *TpSin1* promotes silica formation through synergistic interactions with long-chain polyamines [28]. In this study, the overexpression and knockdown of *CcSAP2* resulted in an increase and decrease of the biosilica content of *C. cryptica*, respectively, implying that *CcSAP2* is also probably involved in silica deposition. However, more research is needed in the next step to provide substantial evidence for this assumption. Additionally, there is no clue available for the explanation of unchanged frustule morphology in the overexpression lines. We wonder if there is an equivalent relationship between *CcSAP2* and microtubules (or microtubule binding sites). The binding status, rather than the amount of specific components alone, determines the formation of the base layer, and thus the unilateral increase of unilateral increase of *CcSAP2 *proteins cannot alter the frustule morphology.

## Conclusion

In this study, we genetically manipulated the microstructure of *C. cryptica* frustules. The *CcSAP2* knockdown strains exhibited a smaller size, altered surface pattern, larger BET, and smaller pore diameter compared to the WT. In these cases, the knockdown strains showed a shorter coagulation time and lower blood loss in an in vivo hemostatic model, suggesting that the altered microstructure enabled better liquid absorption and faster activation of the coagulation cascade. The findings of this study may provide guidance on how to genetically modify the frustule morphology to achieve desired hemostatic characteristics.

### Supplementary Information

**Additional file 1: Figure S1.** Plasmid map of CcpPHa-T1. Bleo: bleomycin resistance; Amp: region encoding ampicillin resistance. Native promoter: 13,652 (g10780.t1) promoter; Native terminator: 13,652 (g10780.t1) terminator. **Figure S2.** **a** *CcSAP*s sequences with highlighted signal peptide (green), RXL site (blue), transmembrane domain (bold font), conserved domain after transmembrane domain (yellow). **b** The schematic of CcSAPs. **Figure S3.** Transcript patterns of *CcSAP1* (a), *CcSAP2* (b), *CcSAP3* (c) under Si-starvation and Si-replete conditions. **Figure S4.** The amplification of the *ble* gene in ten transformation strains. **Figure S5.** The N_2_ adsorption–desorption isotherms and the corresponding pore size distribution of wide type (**a**) and *CcSAP2* knockdown lines of A-2 (**b**), A-4 (**c**), and A-5 (**d**). **Figure S6.** The blood compatibility of frustules (**a**-**d**). WT represents the wild type strain; A-2, A-4, and A-5 represent the antisense RNA clones. **Figure S7.** **a** The linear regression analysis between BET value and MA (mm) of frustules. **b** The linear fit of liquid absorbability and MA (mm) frustules. **Table S1.** Primers used to amplify target genes for cloning.

## Data Availability

All data are available upon request to the corresponding author.
