# Supplementary material for: Enhancement of hemostatic properties of Cyclotella cryptica frustule through genetic manipulation
Source: Biotechnol Biofuels Bioprod. 2023 Sep 14;16:136. doi: 10.1186/s13068-023-02389-x (PMC10503012; doi:10.1186/s13068-023-02389-x)
Supplement: Supplementary file 1 — Additional file 1: Figure S1. Plasmid map of CcpPHa-T1. Bleo: bleomycin resistance; Amp: region encoding ampicillin resistance. Native promoter: 13,652 (g10780.t1) promoter; Native terminator: 13,652 (g10780.t1) terminator. Figure S2. a CcSAPs sequences with highlighted signal peptide (green), RXL site (blue), transmembrane domain (bold font), conserved domain after transmembrane domain (yellow). b The schematic of CcSAPs. Figure S3. Transcript patterns of CcSAP1 (a), CcSAP2 (b), CcSAP3 (c) under Si-starvation and Si-replete conditions. Figure S4. The amplification of the ble gene in ten transformation strains. Figure S5. The N2 adsorption–desorption isotherms and the corresponding pore size distribution of wide type (a) and CcSAP2 knockdown lines of A-2 (b), A-4 (c), and A-5 (d). Figure S6. The blood compatibility of frustules (a-d). WT represents the wild type strain; A-2, A-4, and A-5 represent the antisense RNA clones. Figure S7. a The linear regression analysis between BET value and MA (mm) of frustules. b The linear fit of liquid absorbability and MA (mm) frustules. Table S1. Primers used to amplify target genes for cloning. [file 13068_2023_2389_MOESM1_ESM.docx]

**Enhancement of hemostatic properties of *Cyclotella cryptica* frustule through genetic manipulation**

Lulu Wang^1^, Yan Sun^2^, Ruihao Zhang^1^, Kehou Pan^1,3^, Yuhang Li^4^, Ruibing Wang^5^, Lin Zhang^6^, Chengxu Zhou^2^, Jian Li ^7^, Yun Li^1^, Baohua Zhu^1^, Jichang Han^2*^

1 Key Laboratory of Mariculture, Ocean University of China, Ministry of Education, Qingdao 266003, China

2 College of Food and Pharmaceutical Sciences, Ningbo University, Ningbo 315200, China.

3 Laoshan Laboratory, Qingdao 266237, China

4 Department of Marine Organism Taxonomy and Phylogeny, Institute of Oceanology, Chinese Academy of Sciences, Qingdao 266071, China.

5 State Key Laboratory of Quality Research in Chinese Medicine, Institute of Chinese Medical Sciences, University of Macau, Macau 999078, China.

6 Key Laboratory of Applied Marine Biotechnology, School of Marine Sciences, Ningbo University, Ningbo 315200, China.

7 School of Biological and Chemical Engineering, Panzhihua University, Panzhihua 617000, China.

*Corresponding author: [Hanjichang@nbu.edu.cn](mailto:Hanjichang@nbu.edu.cn)


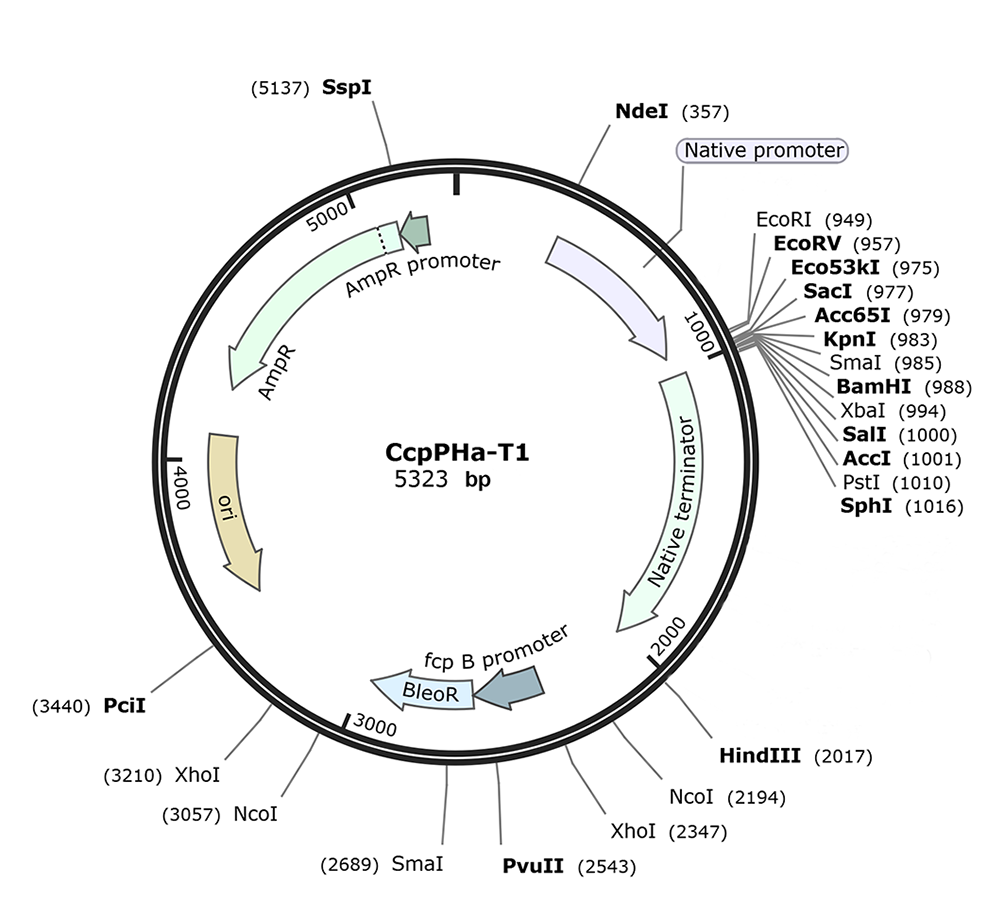


Figure S1. Plasmid map of CcpPHa-T1. Bleo: bleomycin resistance; Amp: region encoding ampicillin resistance. Native promoter: 13652 (g10780.t1) promoter; Native terminator: 13652 (g10780.t1) terminator.


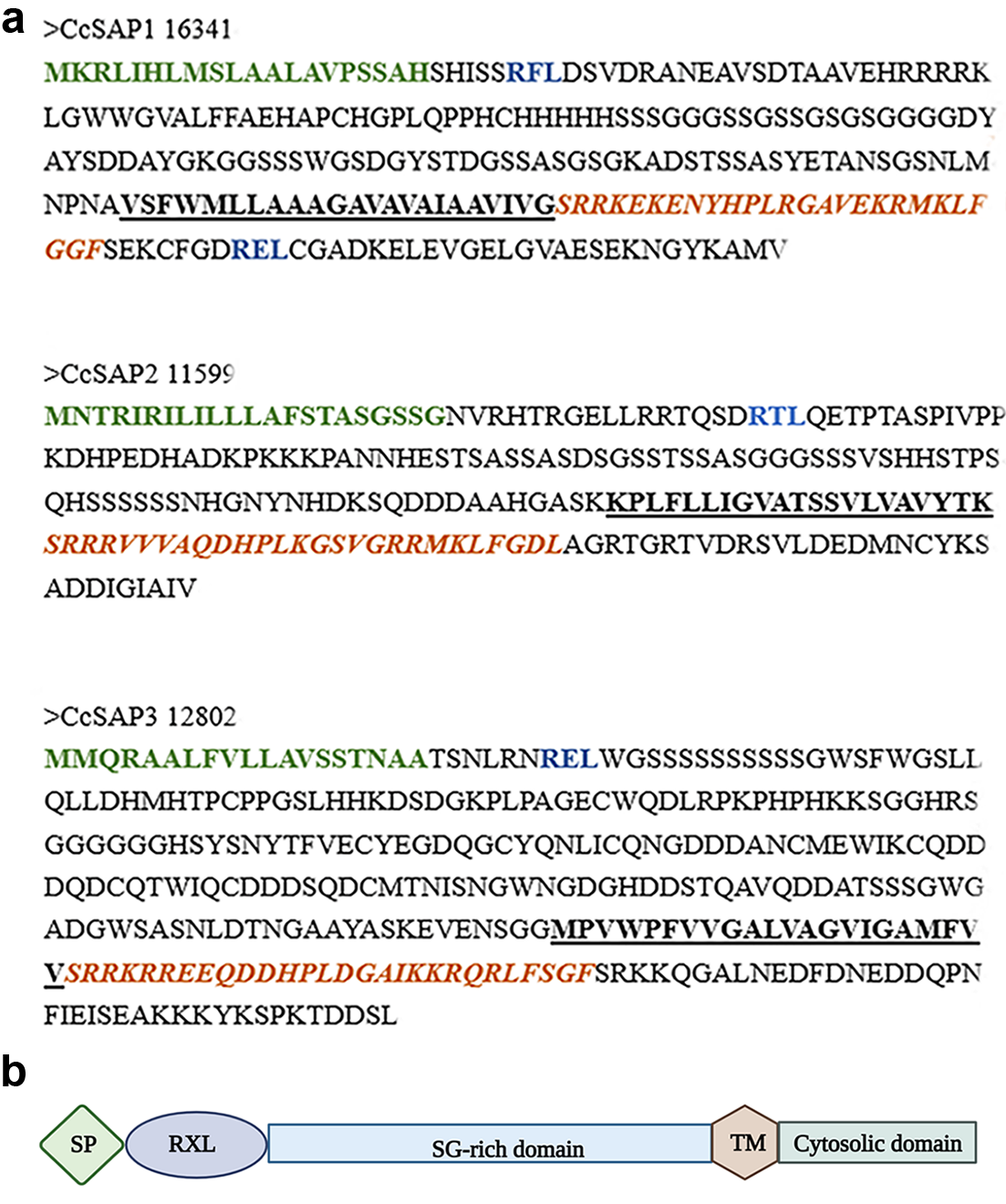


Figure S2. (a) *CcSAP*s sequences with highlighted signal peptide (green), RXL site (blue), transmembrane domain (bold font), conserved domain after transmembrane domain (yellow); (b) The schematic of *CcSAP*s; (c-e) The predicted domain of three *CcSAP*3.


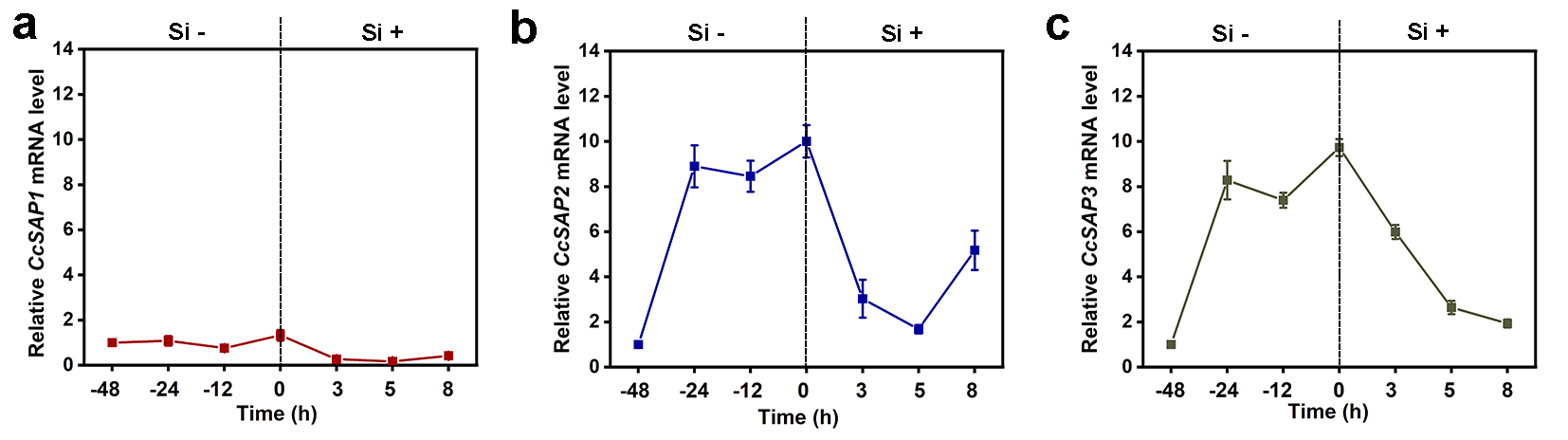


Figure S3. Transcript patterns of *CcSAP1* (a), *CcSAP2* (b), *CcSAP3* (c) under Si-starvation and Si-replete conditions.


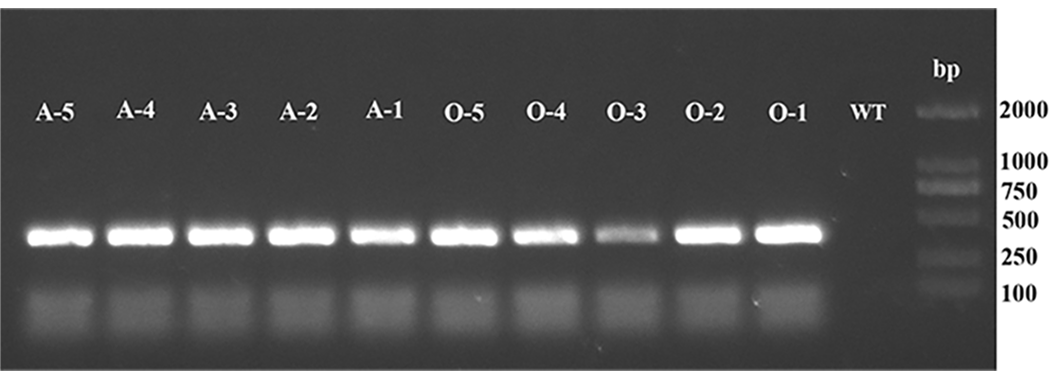


Figure S4. The amplification of the *ble* gene in ten transformation strains.


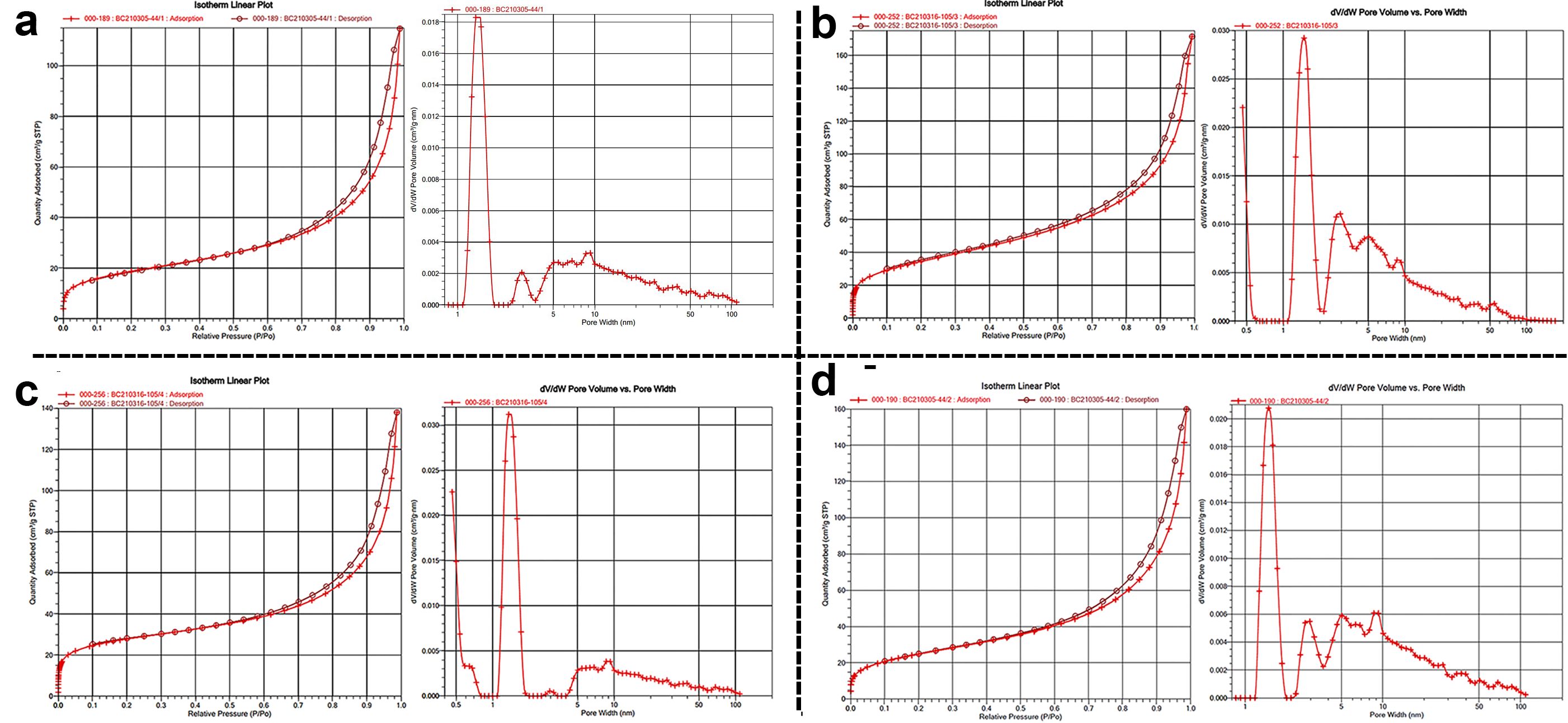


Figure S5. The N_2_ adsorption–desorption isotherms and the corresponding pore size distribution of wide type (a) and *CcSAP2* knockdown lines of A-2 (b), A-4 (c), and A-5 (d).


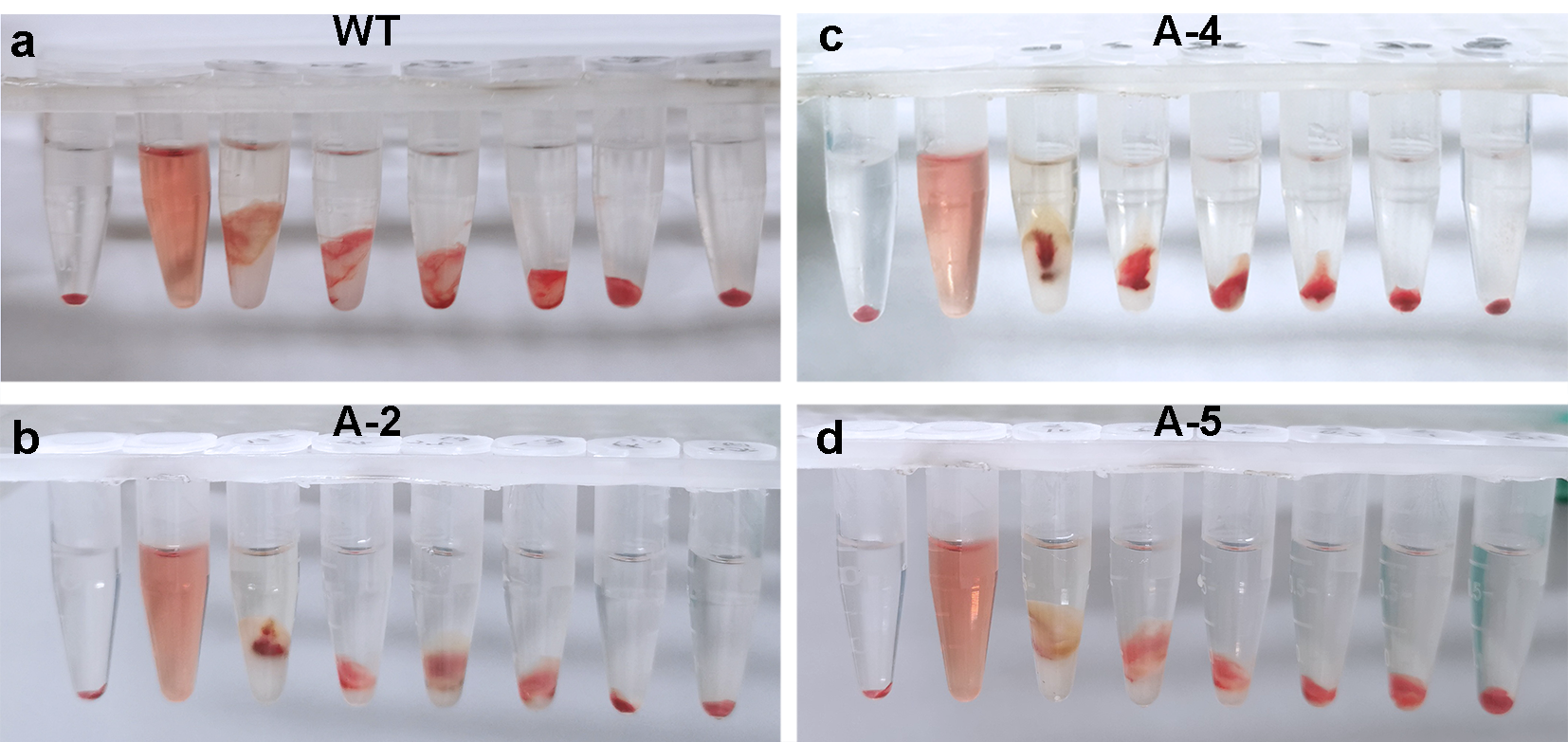


Figure S6. The blood compatibility of frustules (a-d). WT represents the wild type strain; A-2, A-4, and A-5 represent the antisense RNA clones.


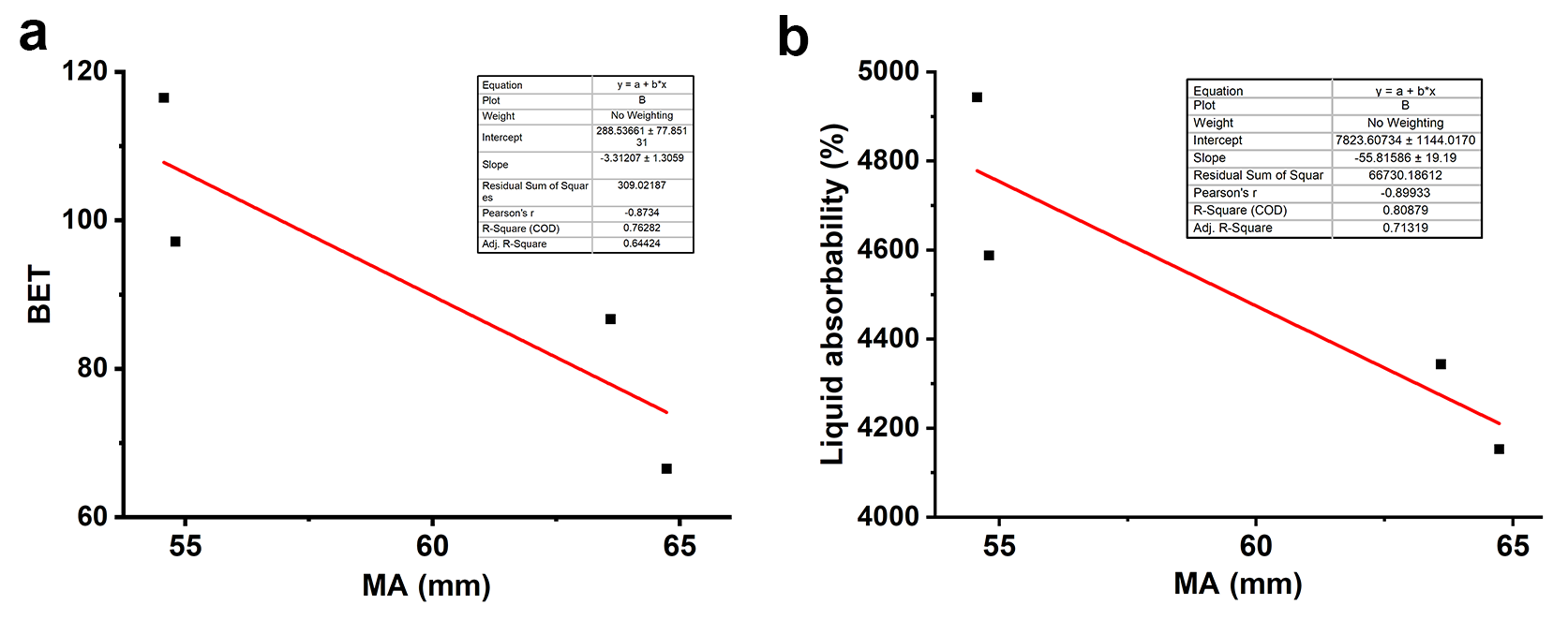


Figure S7. (a) The linear regression analysis between BET value and MA (mm) of frustules; (b) The linear fit of liquid absorbability and MA (mm) frustules.

Table S1. Primers used to amplify target genes for cloning

|  | Name | Sequence |
| --- | --- | --- |
| Native promoter | 587-fw | CCATATGGGTTTCACTTGGTGTCACTCGG |
| Native promoter | 587-rv | GGAATTCGATCGATGTTTGAGTCAGGTTGA |
| Native terminator | 999-fw | GGCATGCGCTCTCAGACTATGAAATGAATAAT |
| Native terminator | 999-rv | CCAAGCTTGCGACTGAAGTGACAACTCTAG |
| Overexpression | *SAP2*-s-fw | CGAGCTCATGAACACCAGAATACGAATACTC |
| Overexpression | *SAP2*-s-rv | CGGGATCCCTAATGATGATGATGATGATGAACAATTGCAATC |
| Knockdown (antisense) | *SAP2*-as-fw | CGGGATCCAACACCAGAATACGAATACTC |
| Knockdown (antisense) | *SAP2*-as-rv | CGAGCTCCTAAACAATTGCAATC |
| Resistance gene | Ble-fw | CACGGTTGCCAGATGTCAAGATGGCCAAG |
| Resistance gene | Ble-rv | GGTTCAGTCCTGCTCCTCGGCCACGAAGTG |
| Reference gene | H4-fw | AAGCGCATCTCTGGGCTCAT |
| Reference gene | H4-rv | AGAGAGTCTTGCCCTGACGC |
| Quantitative PCR | *CcSAP*1-fw | GGAAAGGCGGGAGTTCCAGTT |
| Quantitative PCR | *CcSAP*1-rv | ATGACACAGCGTTCGGGTTCA |
| Quantitative PCR | *CcSAP*2-fw | GAGACGACGATGCCAACTGT |
| Quantitative PCR | *CcSAP*2-rv | GCCCCATCCACTGCTACTTG |
| Quantitative PCR | *CcSAP*3-fw | CCTCCTCTTCCTCCTCCAACCA |
| Quantitative PCR | *CcSAP*3-rv | GACAACCCGTCGACGTGACTT |
